# Polar recruitment of RLD by LAZY1-like protein during gravity signaling in root branch angle control

**DOI:** 10.1038/s41467-019-13729-7

**Published:** 2020-01-03

**Authors:** Masahiko Furutani, Yoshinori Hirano, Takeshi Nishimura, Moritaka Nakamura, Masatoshi Taniguchi, Kanako Suzuki, Ryuichiro Oshida, Chiemi Kondo, Song Sun, Kagayaki Kato, Yoichiro Fukao, Toshio Hakoshima, Miyo Terao Morita

**Affiliations:** 1 College of Life Sciences, Fujian Agriculture and Forestry University, Fuzhou, Fujian 350002 China; 2 FAFU-UCR Joint Center, Fujian Provincial Key Laboratory of Haixia Applied Plant System Biology, Haixia Institute of Science and Technology, Fujian Agriculture and Forestry University, Fuzhou, Fujian 350002 China; 3 Graduate School of Bioagricultural Sciences, Nagoya University, Chikusa, Nagoya 464-8601 Japan; 4 Graduate School of Biological Sciences, Nara Institute of Science and Technology, Ikoma, 630-0101 Japan; 5 Graduate School of Pharmaceutical Sciences, The University of Tokyo, Bunkyo-ku, Tokyo 113-0033 Japan; 6 Division of Plant Environmental Responses, National Institute for Basic Biology, Myodaiji, Okazaki 444-8556 Japan; 7 Laboratory of Biological Diversity, National Institute for Basic Biology, Myodaiji, Okazaki 444-8556 Japan; 8 Bioimage Informatics Group, Exploratory Research Center on Life and Living Systems (ExCELLS), National Institute of Natural Sciences, Myodaiji, Okazaki 444-8585 Japan; 9 Department of Bioinformatics, Ritsumeikan University, Kusatsu, 525-8577 Japan

**Keywords:** Plant polarity, X-ray crystallography

## Abstract

In many plant species, roots maintain specific growth angles relative to the direction of gravity, known as gravitropic set point angles (GSAs). These contribute to the efficient acquisition of water and nutrients. *AtLAZY1/LAZY1-LIKE* (*LZY*) genes are involved in GSA control by regulating auxin flow toward the direction of gravity in Arabidopsis. Here, we demonstrate that RCC1-like domain (RLD) proteins, identified as LZY interactors, are essential regulators of polar auxin transport. We show that interaction of the CCL domain of LZY with the BRX domain of RLD is important for the recruitment of RLD from the cytoplasm to the plasma membrane by LZY. A structural analysis reveals the mode of the interaction as an intermolecular β-sheet in addition to the structure of the BRX domain. Our results offer a molecular framework in which gravity signal first emerges as polarized LZY3 localization in gravity-sensing cells, followed by polar RLD1 localization and PIN3 relocalization to modulate auxin flow.

## Abstract (web summary)

In Arabidopsis, LZY proteins are involved in specifying the angle of root growth relative to the direction of gravity. Here the authors identify RLD1, a LZY-interacting protein that is recruited to the plasma membrane in a polar manner and is required to establish asymmetric auxin transport and set root angles.

## Introduction

Gravity is a fundamental environmental signal that affects all organisms on Earth. Plant organs sense gravity as a directional cue to control their growth orientation and shoots typically grow upward while roots grow downward. This growth response to gravity is known as gravitropism^1–3^. Plant roots and shoots are generally maintained at specific angles relative to the direction of gravity, referred to as the gravitropic setpoint angle (GSA), which is one aspect of gravitropism^4–7^. Proper positioning of leaves or roots enables efficient light reception and reproduction or water and nutrient uptake, respectively. Thus, the regulation of branch angles is an important trait in crop breeding to increase yield^8,9^.

When plant organs incline and turn away from GSA, a change in their orientation relative to gravity is perceived in specialized gravity-sensing cells, known as statocytes. In *Arabidopsis thaliana*, shoot endodermal cells and root columella cells are statocytes that contain high-density starch-accumulating amyloplasts^3,10–12^. Amyloplasts play a role as statoliths; that is, they relocate according to the direction of gravity, triggering intracellular signaling^2^. Subsequently, the signal promotes the transport of the plant hormone auxin toward the direction of gravity in the responsive organ, resulting in the differential growth of the organ. Thus, gravity signaling in statocytes is a key process in which physical information derived from amyloplast sedimentation is converted to regulation of auxin transport^13^. PIN-FORMED3 (PIN3), a member of auxin efflux facilitator PIN family, is uniformly localized to the plasma membrane (PM) of statocytes in vertically growing Arabidopsis organs. Upon gravistimulation by reorientation, PIN3 undergoes polar localization to the lower side (the direction of gravity) of the statocytes of the roots and hypocotyls, which could contribute to the directional transport of auxin to the lower flank of the organs^14–17^. Meanwhile, *LAZY1* family genes are involved in gravitropism in many plant species^13,18–24^. In Arabidopsis, at least four of the six *LAZY1-LIKE* (*LZY*) genes are redundantly required for gravitropism of roots and shoots^13,22^ and *LZY1*, *LZY2*, and *LZY3* play a key role in gravity signaling in statocytes^13^. *PIN3* and *LZY* have also been shown to be involved in the GSA control of lateral roots (LRs)^13,25^, suggesting that a similar gravity signaling mechanism is required for GSA control of lateral organs. In Arabidopsis, temporal regulation of PIN expression during LR elongation, that is, early transient expression of PIN3 and subsequent expression of PIN4 and PIN7 affects the GSA of young LRs^25^. We previously demonstrated that *LZY* genes facilitate polar auxin transport toward the direction of gravity, possibly through the control of asymmetric PIN3 expression in the root cap columella of LRs^13^. However, LZYs are plant-specific unknown proteins with no domain for which the function is inferable. For further understanding of the gravity signaling mechanism, it is essential to elucidate the molecular function of the LZY protein.

Here, we identify RCC1-like domain (RLD) proteins as LZY interactors and reveal that RLD is a regulator of polar auxin transport that controls the abundance and localization of the PIN protein in various developmental processes including GSA control. Structural analysis of the complex of CCL domain of LZY and BRX domain of RLD, which are responsible for the direct interaction of these proteins, reveals hydrophobic and electrostatic interactions at the interface of the anti-parallel intermolecular β-sheet. Furthermore, we find that LZY3 localization is polarized in the direction of gravity in the PM of columella cells of LRs upon gravistimulation, resulting in polar recruitment of RLD1 to the PM of the cell as well as PIN3 relocalization. We propose a model of gravity signaling involving the modulation of auxin flow in LR columella cells by LZY and RLD.

## Results

### RLDs are involved in GSA control

**Fig. 1:**
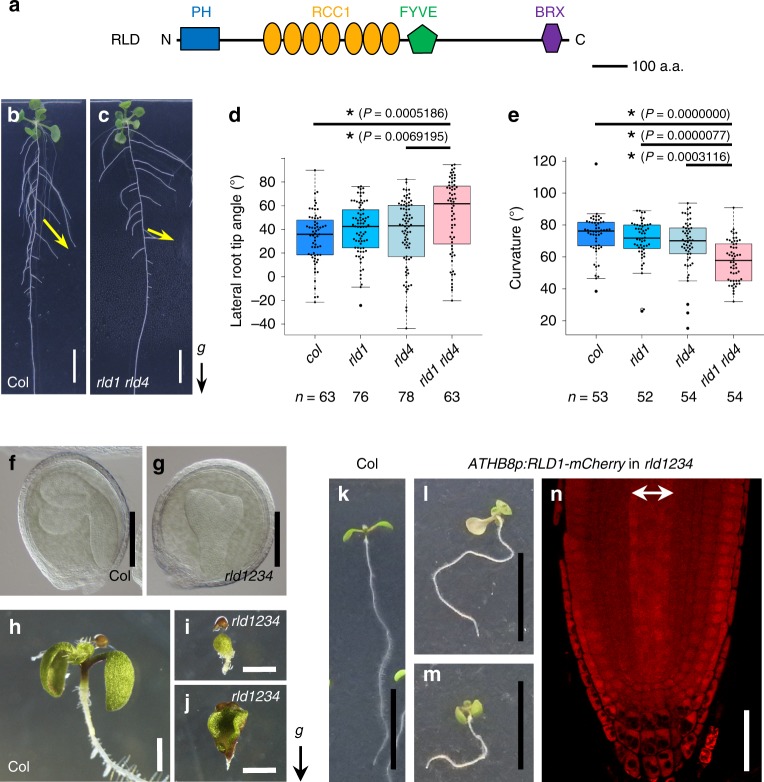
*RLD* genes regulate GSA in LRs and embryo development. **a** Schematic diagram of RLD. **b**, **c** Twelve-day-old seedlings of Col (**b**) and *rld1-2 rld4-1* double mutant (**c**). Arrows indicate the growth orientation of lateral roots. **d** Lateral root tip angle is represented by boxplots for 12-day-old seedlings of Col, *rld1-2*, *rld4-1*, and *rld1-2 rld4-1* toward the direction of gravity. Median and quartile values are provided by the central line and box boundaries. Whiskers show min to max values. *n*, sample number of three biologically independent experiments. Asterisks indicate significant differences by the Tukey–Kramer method (*P* < 0.05). **e** Comparison of root curvature of 5-day-old seedlings of each line at 12 h after a 90° reorientation. *n*, sample number of three biologically independent experiments. Asterisks show significant differences by the Tukey–Kramer method (*P* < 0.05). **f**, **g** Embryos of Col (**f**) and *rld1-2 rld2-2 rld3-2 rld4-1* (**g**). **h**–**j** Seven-day-old seedlings of Col (**h**) and *rld1-2 rld2-2 rld3-2 rld4-1* (**i**, **j**). **k**–**m** Seven-day-old seedlings of Col (**k**) and partial complemented *rld1-2 rld2-2 rld3-2 rld4-1* seedlings by expressing *RLD1*-*mCherry* under control of the *ATHB8* promoter (**l**, **m**). **n** RLD1-mCherry expression in the primary root of 5-day-old *rld1-2 rld2-2 rld3-2 rld4-1* seedling harboring *ATHB8p:RLD1*-*mCherry*. A double-head arrow indicates RLD-mCherry expression domain in the vasculature. Arrow marked with “*g*” represents the direction of gravity. Scale bars, 1 cm (**b**, **c**), 100 μm (**f**, **g**), 5 mm (**h**–**j**), 2 cm (**k**–**m**), and 50 μm (**n**). Source data for **d**, **e** are provided as a Source Data file.

To clarify the molecular function of LZYs, we identified the proteins that interact with them using yeast two-hybrid screening and immunoprecipitation (IP) coupled with mass spectrometry. We found four out of eight RLD family proteins to be candidates for interaction with LZY2 and LZY3 in both screening strategies (Supplementary Fig. 1; Supplementary Tables 1 and 2). The RLD family proteins are conserved among land plants and share a similar domain combination containing a pleckstrin homology (PH) domain, regulator of chromosome condensation 1 (RCC1)-like motif repeats, a Fab1/YGL023/Vps27/EEA1 (FYVE) domain, and a Brevis radix (BRX) domain^26^ (Fig. 1a). *RLD1* (At1g76950), *RLD2* (At5g12350), *RLD3* (At5g19420), and *RLD4* (At5g42140) were expressed in root caps and vascular tissues of primary roots (PRs) and young LRs (Supplementary Fig. 2). Although GUS activity was scarcely detected in young LRs of *RLD4p:GUS*, the transcript of *RLD4* is detectable during LR development according to a publicly available database, Arabidopsis eFP browser. To test whether *RLD* genes are involved in GSA control of LRs, *rld* mutants were isolated (Supplementary Figs. 3 and 4). While no single mutants exhibited an obvious phenotype (Supplementary Fig. 5), LR tip angles of *rld1 rld4* double mutant were wider than those of the wild type (Fig. 1b–d). The results demonstrated that at least *RLD1* and *RLD4* are involved in GSA control of LRs. The GSA phenotype of *rld1 rld4* LRs was rescued by expressing *RLD1-GFP* under the control of its own promoter *RLD1p* and the statocyte-specific promoter of *ACTIN DEPOLYMERIZING FACTOR9* (*ADF9*)^13^, indicating that functional RLD1-GFP in the root statocytes is responsible for the GSA control of LRs (Supplementary Fig. 6). In addition, the PRs of the *rld1 rld4* double mutant displayed reduced gravitropic responses (Fig. 1e). These phenotypes were mild, suggesting that remaining *RLD* genes, *RLD2* and *RLD3*, could function in GSA control and root gravitropism. To investigate this possibility, a *rld1 rld2 rld3 rld4* quadruple mutant was constructed. Severe defects in organ formation were observed in *rld1 rld2 rld3 rld4* quadruple mutant embryos and seedlings (Fig. 1f–j). Since it is considered that these severe phenotypes of the quadruple mutant are due to impaired vascular development where the promoter activity of *RLD* genes was detected (Supplementary Fig. 2), vascular-specific complementation analysis was performed. The expression of *RLD1-mCherry* driven by the provascular- and vascular-specific promoter of *ARABIDOPSIS THALIANA HOMEOBOX8* (*ATHB8*)^27^ rescued embryo development and root formation in *rld1 rld2 rld3 rld4* seedlings as expected, but it was not sufficient for gravitropic growth of PRs (Fig. 1k–n), suggesting that *RLD2* and *RLD3* are also required for root gravitropism. Our data suggest that *RLD1*–*4* are redundantly involved in GSA control of LRs and root gravitropism in columella cells.

### RLD regulates PIN-dependent auxin transport

**Fig. 2:**
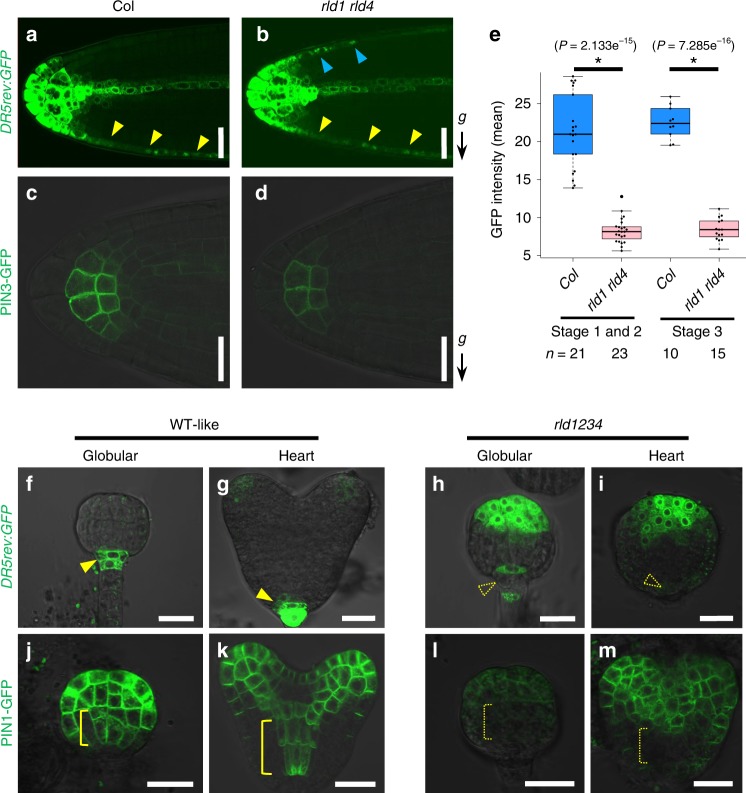
*RLD* genes regulate auxin transport through the control of PIN localization. **a**–**d** *DR5rev:GFP* (**a**, **b**) and PIN3-GFP (**c**, **d**) expression in lateral root tips of Col (**a**, **c**) and *rld1-2 rld4-1* (**b**, **d**). Arrowheads indicate *DR5rev:GFP* expression in lateral root cap cells (upper side, blue; lower side, yellow). **e** GFP intensity in the central columella cells of lateral roots of Col and *rld1-2 rld4-1* harboring *PIN3p:PIN3-GFP*. Median and quartile values are provided by the central line and box boundaries. Whiskers show min to max values. *n*, sample number of three biologically independent experiments. Asterisks indicate significant differences by Student’s *t* test (*P* < 0.05). Source data are provided as a Source Data file. **f**–**m** *DR5rev:GFP* (**f**, **g**, **h**, **i**) and PIN1-GFP (**j**, **k**, **l**, **m**) expression in WT-like (**f**, **g**, **j**, **k**) and *rld1-2 rld2-2 rld3-2 rld4-1* (**h**, **i**, **l**, **m**) embryos dissected from ovules of plant homozygous for *rld1-2*, *rld3-2*, and *rld4-1* and heterozygous for *rld2-2* at the globular (**f**, **h**, **j**, **l**) and heart stage (**g**, **i**, **k**, **m**). Arrowheads and brackets indicate strong GFP signals in the hypophysis/radicle tip and provasculature of basal region, respectively, while blank arrowheads and dashed brackets represent disappeared GFP signals in respective regions. Arrow marked with “*g*” represents the direction of gravity. Scale bars, 20 μm (**a**–**d**, **f**–**m**).

An asymmetric expression pattern of the auxin-responsive promoter driving GFP (*DR5rev:GFP*)^28^ toward the direction of gravity was distinctly observable in wild-type stage 3 LRs^13,25^, whereas it was hardly observed in *rld1 rld4* (Fig. 2a, b). This result indicates that *RLD1* and *RLD4* regulate LR GSA through the control of auxin flow. Loss of function of *RLD1* and *RLD4* caused a significant reduction in the signal intensity of PIN3-GFP in statocytes of the LRs throughout stages 1, 2, and 3 (Fig. 2c–e). In addition, *rld1 rld2 rld3 rld4* mutant embryos displayed severe reduction of PIN1-GFP expression together with defective expression pattern of *DR5rev:GFP* (Fig. 2f–m). These results suggest that *RLD1*–*4* modulates auxin transport through regulation of PIN localization in the GSA control of LRs and during embryogenesis. Striking morphological defects with aberrant expression of PIN1 and *DR5rev:GFP* in *rld* quadruple mutant embryos closely resemble those of *gnom* mutants^29–31^. *GNOM* encodes a brefeldin A (BFA)-sensitive GDP/GTP exchange factor (GEF) for small G proteins of the ADF ribosylation factor (ARF) class. ARF-GEF GNOM-dependent membrane trafficking was previously reported to regulate root gravitropism through the control of PIN3-dependent auxin flow^16^. To examine the relationship between *RLD* genes and ARF-GEF GNOM, pharmacological analysis using BFA, an inhibitor of ARF-GEF, was performed (Supplementary Fig. 7). Without BFA treatment, there were no differences between the wild type and *rld1 rld4* in PR and LR formation. On the other hand, the *rld1 rld4* double mutant displayed BFA-sensitive phenotypes in PR length and in the growth direction of PR (Supplementary Fig. 7a–h). In addition, the 1 μM BFA treatment had a greater negative impact on LR development in *rld1 rld4* than in the wild type (Supplementary Fig. 7b, e, i, j). These findings suggest that the *RLD*s could regulate auxin flow in the same pathway as GNOM to control PIN proteins not only in root gravitropism but also in plant development.

### LZY recruits RLD to the PM

**Fig. 3:**
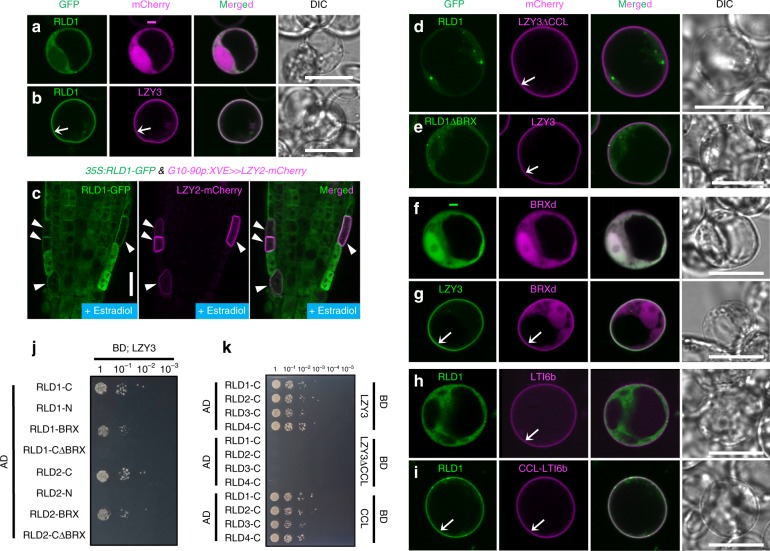
LZY recruits RLD to the plasma membrane via the CCL-BRX interaction. **a**, **b** Co-expression of RLD1-GFP with mCherry (**a**) and LZY3-mCherry (**b**) in Arabidopsis protoplast cells. Arrows indicate plasma membrane-localized signals. **c** Localization of RLD1-GFP (left) and LZY2-mCherry (middle) in primary root of 8-day-old seedling harboring *35S:RLD1*-*GFP* and *G10-90p:XVE»LZY2*-*mCherry*, transferred to MS medium plate containing 1 μM estradiol at 7 days old. Merged image of RLD1-GFP and LZY2-mCherry (right). Arrowheads indicate LZY2-mCherry-expressing cells, where RLD1-GFP was co-localized with LZY2-mCherry in the plasma membrane. **d**, **e** Co-expression of RLD1-GFP and CCL-deleted LZY3-mCherry (LZY3ΔCCL) (**d**), and of the BRX domain-deleted RLD1-GFP (RLD1ΔBRX) and LZY3-mCherry (**e**) in Arabidopsis protoplast cells. **f**, **g** Co-expression of the BRX domain (BRXd)-mCherry with GFP (**f**) and LZY3-GFP (**g**) in Arabidopsis protoplast cells. **h**, **i** Co-expression of RLD1-GFP with mCherry-LTI6b (**h**) and CCL-mCherry-LTI6b (**i**) in Arabidopsis protoplast cells. Scale bars, 10 μm (**a**, **b**, **d**–**i**) and 20 μm (**c**). **j**, **k** Interaction between LZY3 and truncated forms of RLD1 and RLD2 (**j**), and between C terminus of RLDs and truncated forms of LZY3 (**k**) in the Y2H system. Interaction was indicated by growth on selection medium lacking leucine, tryptophan, histidine, and adenine.

Previous studies showed that LZY proteins were localized mainly in the PM of protoplast cells^13^. In contrast, RLD proteins were not localized in the PM of protoplast cells, but in the cytoplasm and punctate structures (Fig. 3a and Supplementary Fig. 8a–c). Interestingly, when co-expressed with LZY2 or LZY3 in protoplast cells, RLD proteins were localized in the PM along with LZY proteins, indicating that LZYs recruit RLDs from the cytoplasm and punctate structures to the PM (Fig. 2b and Supplementary Fig. 8d–h). To confirm LZY-dependent RLD recruitment to the PM in plants, LZY2-mCherry and RLD1-GFP were co-expressed in seedlings harboring both *35S:RLD1-GFP* and *G10-90p:XVE»LZY2-mCherry*. In root cap cells, ectopically expressed RLD1-GFP was localized in the cytoplasm (Fig. 3c). When co-expressed with LZY2-mCherry, the RLD1-GFP signal was detected mainly in the PM. Next, the domains of both LZY and RLD responsible for LZY-dependent RLD recruitment to the PM were identified, using transient assays in protoplast cells. The CCL domain of LZY and the BRX domain of RLD were required for the recruitment of RLD to the PM by LZYs (Fig. 3d, e, Supplementary Fig. 8i, j, and Supplementary Fig. 9e). In addition, the BRX domain is sufficient for LZY3-dependent PM localization in protoplasts and statocytes (Fig. 3f, g and Supplementary Fig. 10) and the CCL domain is sufficient to recruit RLD1 to the PM when fused with the PM protein LTI6b (Fig. 3h, i). Furthermore, we found that the CCL domain of LZY3 and the BRX domain of all RLDs are necessary and sufficient for the interaction in yeast cells (Fig. 3j, k and Supplementary Fig. 11). To confirm the direct interaction between the CCL domain and the BRX domain, we performed in vitro pull-down binding assay. Since the amino acid sequence of the CCL domain of LZY2 and LZY3 is the same, we used the CCL domain of LZY1 and LZY3 (Supplementary Fig. 12). Binding between the CCL and the BRX was detected in all combinations of LZYs and RLDs. These results demonstrate that direct interaction between the CCL domain and the BRX domain is responsible for the LZY-RLD interaction, which is necessary and sufficient for the recruitment of RLD from the cytosol to the PM.

### Structural analysis of the CCL-BRX complex

**Fig. 4:**
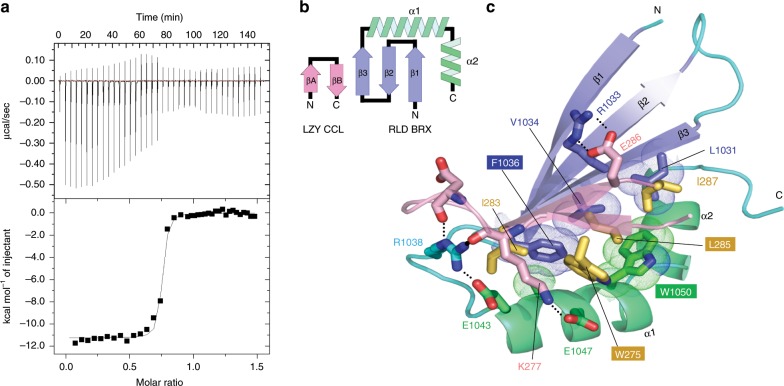
The structure of the CCL-BRX complex. **a** Binding of the RLD2 BRX domain to the LZY3 CCL peptide. The obtained ITC profile showed formation of a 1:1 complex with a small *K*_D_ value (9.7 ± 2.2 nM). *ΔH*, *TΔS*, and *N* are −11.3 ± 0.09 kcal/mol, −1.68 kcal/mol, and 0.73 ± 0.002, respectively. **b** Topology diagram of the RLD2 BRX domain bound to LZY3 CCL. **c** Ribbon representation of the crystal structure of the BRX-CCL complex. Color codes are as in **b**. The RLD2 BRX domain comprises a three-stranded antiparallel β-sheet (blue) and two α-helices (green). The LZY3 CCL peptide adopts a β-hairpin structure (pink). Dashed lines represent inter-molecular hydrogen bonds. The colors of corresponding positions in the CCL-BRX complex at which mutations were introduced in RLD1-GFP and LZY3-mCherry are reversed.

**Fig. 5:**
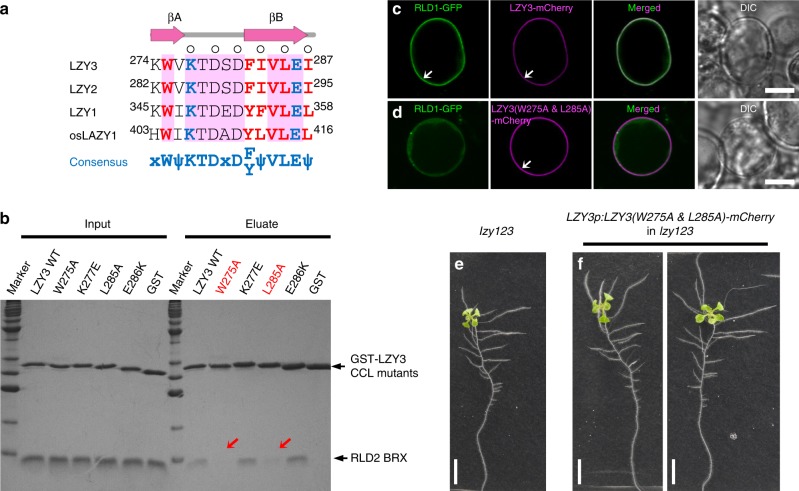
The disruptive mutations of the CCL-BRX interaction interfered with LZY3 function. **a** Sequence alignment of LZYs CCL with the secondary structures are shown at the top with β-strands (arrowhead). The conserved sequences are filled in pink. Residues whose side chain atoms form inter-molecular hydrogen bonds and residues making intermolecular van der Waals contacts are colored in cyan and red, respectively, while residues whose main chain form inter-molecular hydrogen bonds are indicated with open circle. In the consensus sequence, Ψ represents a hydrophobic residue, while x represents any residue. **b** Pull-down binding assay. RLD2 BRX domain was pulled down with GST-LZY3 CCL (WT and mutants) and analyzed by SDS-PAGE. Arrows indicate the reduced abundance of RLD2 BRX. **c**, **d** The importance of CCL-BRX interaction in LZY3-dependent RLD recruitment to the plasma membrane. Co-expression of RLD1-GFP with wild-type LZY3-mCherry (**c**) and LZY3-mCherry carrying mutations in CCL (**d**) in Arabidopsis protoplast cells. **e**, **f** Complementation test. Ten-day-old seedlings of *lzy1 lzy2 lzy3* (**e**) and *lzy1 lzy2 lzy3* uncomplemented by *LZY3-mCherry* carrying mutations in CCL driven by control of the *LZY3* promoter (**f**). Scale bars, 10 μm (**c**, **d**) and 1 cm (**e**, **f**).

To characterize the interaction between RLDs and LZY3, the RLD2 BRX domain and the LZY3 CCL peptide were purified and subjected to a binding assay using isothermal titration calorimetry (ITC). We found that the BRX domain and the CCL peptide form a 1:1 complex with a *K*_D_ value of 9.7 nM (Fig. 4a). To elucidate the structural basis of the interaction, we determined the crystal structures of the RLD2 BRX domain bound to the LZY3 CCL peptide (referred to as the RLD2-LZY3 complex) at 1.35 Å resolution (Supplementary Table 3). The RLD2 BRX domain adopts a compact α + β structure comprising an N-terminal three-stranded antiparallel β-sheet and C-terminal α1- and α2-helices (Fig. 4b, c). The LZY3 CCL peptide is folded into a β-hairpin structure and docks into the hydrophobic groove between the β3-strand and the α1-helix of the RLD2 BRX domain by forming an antiparallel intermolecular β–β association between the βB-strand of the CCL β-hairpin and the β3-strand of the BRX domain. This results in structural extension from the three-stranded β-sheet of the BRX domain to a five-stranded antiparallel β-sheet in the complex, which creates an additional hydrophobic core comprising nonpolar residues (Trp275, Ile283, Leu285, and Ile287) from the CCL β-hairpin and the surface nonpolar residues (Leu1031, Val1034, Phe1036, and Trp1050) from the BRX domain (Fig. 5a and Supplementary Figs. 13 and 14). The complex is also stabilized by polar interactions and the electrostatic surface potential of the BRX domain is complementary to charged residues of the CCL hairpin (Supplementary Fig. 14a). At the interface, salt bridges between charged side chains from the CCL hairpin and the BRX domain are formed (Glu286-Arg1033 and Lys277-Glu1047), and the CCL hairpin loop is stabilized by Arg1038 from the BRX domain (Supplementary Fig. 14b). Our mutational analysis suggests a dominant contribution of hydrophobic interactions to the complex formation and the importance of Arg1038-mediated stabilization of the hairpin loop (Fig. 5b and Supplementary Fig. 14c, d). Using transient expression in protoplast cells, mutations at corresponding Phe1052 and Trp1066 of the RLD1 BRX domain or at Trp275 and Leu285 of LZY3 CCL were found to prevent the recruitment of RLD1 to the PM by LZY3 (Fig. 5c, d and Supplementary Figs. 14e, f, 15). These results demonstrate that the CCL-BRX interaction is sufficient for LZY-dependent RLD recruitment to the PM. In addition, to evaluate the importance of the CCL-BRX interaction in GSA control of LRs, the same mutations were introduced into LZY3 CCL at Trp275 and Leu285 and mutated *LZY3-mCherry* was expressed in the *lzy1 lzy2 lzy3* triple mutants with wider GSA of LRs under the control of own promoter. As expected, mutated LZY3-mCherry failed to complement the triple mutant phenotypes (Fig. 5e, f), demonstrating the significance of the CCL-BRX interaction in GSA control of LRs. Statocyte-specific expression of the RLD2 BRX domain caused wider LR growth angles not only in the wild type but also in the *rld1 rld4* mutant (Supplementary Fig. 16a–f). Moreover, the same construct caused negative root gravitropism in the *lzy1 lzy2 lzy3* background (Supplementary Fig. 16g–k), which was similar to the effect of CCL on *lzy1 lzy2 lzy3* roots^13^. These phenotypes could result from a reduction of the activity of residual LZY and RLD in columella cells by blocking LZY-RLD interaction. These results also support the role of direct CCL-BRX binding in gravity signaling in root statocytes.

### Polar recruitment of RLD by LZY in the direction of gravity

**Fig. 6:**
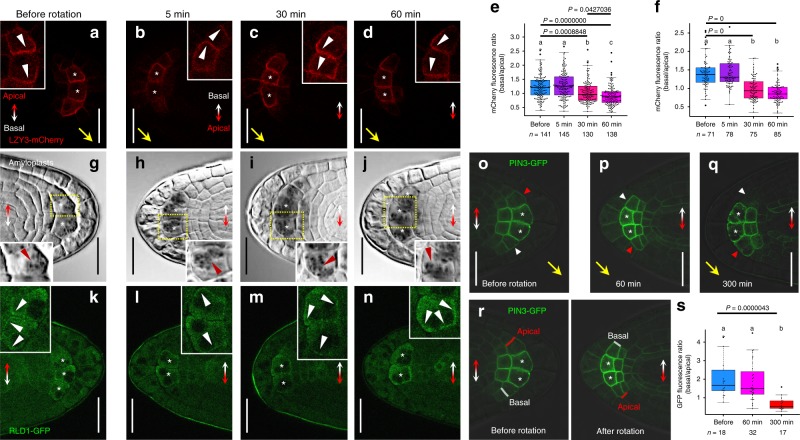
Asymmetric localization of LZY3-mCherry, RLD1-GFP, and PIN3-GFP to the direction of gravity. **a**–**d** The localization of LZY3-mCherry in the LR tips of 8-day-old *lzy1 lzy2 lzy3* seedlings harboring *LZY3p:LZY3-mCherry* at the stage 2 before rotation (**a**) and at 5 min (**b**), 30 min (**c**), and 60 min (**d**) after 180° rotation. White and yellow arrowheads indicate polarized LZY3-mCherry localization and the direction of gravity estimated from the growth orientation of LR tips, respectively. Double-headed arrows show the apical-basal axis of the plant body. Asterisks indicate central columella cells. **e**, **f** Comparison of asymmetric LZY3-mCherry localization in columella cells (**e**) and central columella cells (**f**) in LR tips at stage 2 before and after 180° rotation. Median and quartile values are provided by the central line and box boundaries. Whiskers show min to max values. **g**–**j** The position of amyloplasts in columella cells of LRs of 8-day-old *lzy1 lzy2 lzy3* seedlings harboring *LZY3p:LZY3-mCherry* before and after 180° rotation. Brown arrowhead indicates a biased distribution of amyloplasts in columella cells. **k**–**n** The localization of RLD1-GFP in LR tips of 8-day-old seedlings harboring *RLD1p:RLD1-GFP* before and after 180° rotation. **o**–**q** The localization of PIN3-GFP in the LR tips of 8-day-old seedlings harboring *PIN3p:PIN3-GFP* before and after 180° rotation. Arrowheads indicate lateral columella cells, adjacent to central columella cells (red, apical flank; white, basal flank). **r** Measurement of PIN3-GFP fluorescence intensity at lateral plasma membrane domains of columella cells adjacent to central columella cells at the basal flanks (white) compared with those at the apical flanks (red) in LR tips of 8-day-old seedlings harboring *PIN3p:PIN3-GFP*. **s** Comparison of asymmetric localization of PIN3-GFP in the LR tips of 8-day-old seedlings harboring *PIN3p:PIN3-GFP* before and after 180° rotation. *n*, sample number of three biologically independent experiments (**e**, **f**, **s**). Different letters in **e**, **f**, **s** indicate statistical differences (Tukey–Kramer, *P* < 0.05). Source data for **e**, **f**, and **s** are provided as a Source Data file. Scale bars, 20 μm.

Next, we analyzed LZY3 localization in root statocytes using the complemented transgenic line *LZY3p:LZY3-mCherry*^13^ (Supplementary Fig. 17c, d). Although fluorescence of LZY3-mCherry was not detectable in living roots as previously reported^13^ (Supplementary Fig. 18a, b), the signal became observable in over 80% of LRs (48 out of 58 LR tips in stages 2 and 3) by fixation and clearing with ClearSee solution^32^ (Fig. 6a and Supplementary Fig. 18c–h). In the stage 2 LRs, LZY3-mCherry was detected in the second-outermost layer of the root tips and mainly localized to the PM. Interestingly, LZY3-mCherry was polarly localized in the lower side of the PM (Fig. 6a and Supplementary Fig. 19). To test whether the polarity was determined based on the apical–basal axis of plant body or the direction of gravity, we analyzed LZY3 localization in response to gravistimulation by 180° reorientation experiments. In columella cells of the stage 2 LRs, the mCherry signal was still on the basal side of PM just 5 min after reorientation, while it was detectable in the apical side after 30 min (Fig. 6b–f). The polarity of LZY3-mCherry in the direction of gravity was clearer at 60 min after reorientation than after 30 min (Fig. 6e). In central columella cells, LZY3-mCherry was fully repolarized in the PM at 30 min after reorientation (Fig. 6f). These results indicate that the polarity of LZY3-mCherry localization is determined based on the direction of gravity. Given that the directional change of gravity is thought to be perceived as relocation of amyloplasts in the statocytes, amyloplasts were analyzed by staining starch granules. In the stage 2 LRs, starch granules were visible at the lower side (gravity direction) of the columella cells (Fig. 6g). After the 180° rotation of seedlings, their relocation to the direction of gravity was observed after 30 min of reorientation (Fig. 6h–j), consistent with the timing of relocalization of LZY3-mCherry. These results show a strong correlation between LZY3 polarization and amyloplast sedimentation in columella cells. Next, to test whether RLDs are also polarly localized to the PM in columella cells, intracellular localization of RLD1-GFP in the stage 2 LRs harboring *RLD1p:RLD1-GFP* was observed. GFP fluorescence was predominantly found in the cytoplasm and slightly higher signals were occasionally observed at the cell periphery, but without distinct polarity under normal conditions (Fig. 6k). Interestingly, the localization of RLD1-GFP in the PM was polarized toward the direction of gravity after 60 min of reorientation (Fig. 6l–n), the time when repolarization of LZY3-mCherry localization became obvious (Fig. 6d). Asymmetric localization of PIN3-GFP toward the direction of gravity was previously reported in columella cells of LRs^13,25^. Next, to investigate the gravity-responsive localization of PIN3 and the function of *RLD*s in the PIN3 response, a localization analysis of PIN3-GFP was performed in the stage 2 wild type and *rld1 rld4* LRs (Fig. 6o–q and Supplementary Fig. 20). Since the polarity of PIN3-GFP localization was scarcely detectable in the PM of the columella cells in the LRs, we measured PIN3-GFP fluorescence intensity at the outer lateral PM domains of the lateral columella cell adjacent to the central columella cells and determined the asymmetry of PIN3-GFP as the ratio of the intensity at the basal flank to that at the apical flank (Fig. 6r). In the columella cells of stage 2 wild-type LRs in the second-outermost layer, asymmetry of PIN3-GFP toward the direction of gravity was observed (Fig. 6o, s). The change in the asymmetry of PIN3-GFP was detected at 300 min after reorientation, but not at 60 min (Fig. 6p, q, s), which was delayed from the timing of the amyloplast relocation, LZY3-mCherry, and RLD1-GFP. In contrast, asymmetry of PIN3-GFP localization in the stage 3 *rld1 rld4* LRs was reduced compared with that in wild-type LRs (Supplementary Fig. 20j). After 180° rotation of the seedlings, the shift of asymmetry of PIN3-GFP to the direction of gravity was decreased in *rld1 rld4* LRs at stages 2 and 3, whereas no asymmetry of PIN3-GFP was detectable in either wild-type or *rld1 rld4* stage 1 LRs (Supplementary Fig. 20 h–j). These results indicate that *RLD*s are involved in the shift of asymmetry of PIN3-GFP to the direction of gravity following amyloplast sedimentation and polarization of LZY3 and RLD1 localization.

**Fig. 7:**
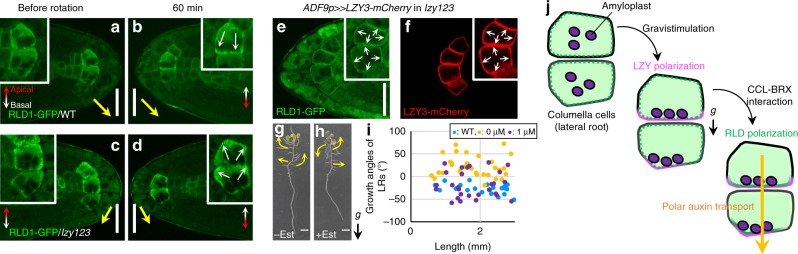
LZYs control RLD1 localization in columella cells of LRs. **a**–**d** LZY-dependent RLD localization to the gravity direction. The RLD1-GFP localization in LRs of 8-day-old Col (**a**, **b**) and *lzy1 lzy2 lzy3* (**c**, **d**) seedlings harboring *RLD1p:RLD1-GFP* before rotation (**a**, **c**) and at 60 min (**b**, **d**) after 180° rotation. White arrows indicate asymmetric localization of RLD1-GFP. Yellow arrows indicate the direction of gravity predicted from the growth angle of LRs vertically grown or after rotation. Double-headed arrows show apical–basal axis of plant body. **e**, **f** Nonpolar localization of RLD1-GFP (**e**) and LZY3-mCherry (**f**) in columella cells of LRs of 8-day-old *lzy1 lzy2 lzy3* seedlings harboring *RLD1p:RLD1-GFP* and *ADF9p:XVE»LZY3-mCherry*, treated with 1 μM estradiol for 48 h, at 60 min after 180° rotation. **g**, **h** Twelve-day-old seedling harboring *ADF9p:XVE»LZY3-mCherry* without (**g**) and with the treatment with 1 μM estradiol (**h**). Yellow arrows indicate the direction of LR growth. **i** Scatterplots of length and growth angle of LRs (<3.0 mm) of 10-day-old plants, Col (blue), *lzy1 lzy2 lzy3* harboring *ADF9p:XVE»LZY3-mCherry* after treatment with EtOH (orange) and 1 μM estradiol (purple) at 4 days. Source data from three biologically independent experiments are provided as a Source Data file. **j** Schematic diagrams of LZY-RLD-mediated gravity signaling in columella cells of LR. Scale bars, 20 μm (**a**–**f**) and 1 cm (**g**, **h**).

In order to investigate the relationship between LZY3 and RLD1 in the statocytes, the localization of LZY3-mCherry and RLD1-GFP was analyzed in the *rld1 rld4* and *lzy1 lzy2 lzy3* mutant background, respectively. The polarization of LZY3-mCherry normally occurred in columella cells of *rld1 rld4* LRs (Supplementary Fig. 21). In contrast, polarized localization of RLD1-GFP to the direction of gravity was not observed at 60 min after reorientation in the *lzy1 lzy2 lzy3* background, but rather the localization of RLD1-GFP was sometimes polarized in the opposite direction of gravity (Fig. 7a–d). These results demonstrate that LZYs recruit RLD1 to the PM on the lower side of the PM in columella cells. Next, to investigate the significance of RLD polarization in GSA control of LRs, LR growth angle was measured when the polarity of RLD localization was disrupted in columella cells. When *LZY3-mCherry* was overexpressed in columella cells, LZY3-mCherry and RLD1-GFP were localized all over the PM, displaying no polarity (Supplementary Fig. 22a, b). In addition, this localization pattern of the two proteins displayed no polarity even after 180° reorientation (Fig. 7e, f). The overexpression of *LZY3-mCherry* induced random growth angles of LRs (Fig. 7g–i and Supplementary Fig. 22c), demonstrating that the polarization of RLD localization plays a key role in GSA control of LRs.

## Discussion

Sequence-based in silico analyses have reported nine FYVE-domain containing proteins including RLD family members as class V AtFYVE proteins in Arabidopsis^33^. We termed eight of the class V AtFYVE proteins harboring a BRX domain at the C-termini as RLD1–8 (Supplementary Fig. 1). RLD family proteins share similar domain architecture, that is, a PH domain followed by six or seven RCC1-like repeats, a FYVE domain, and a BRX domain. RLD family proteins belong to the RCC1 superfamily^34^, which is characterized by a 350–500 residue domain, known as the RLD. Jensen et al.^35^ have reported that the RLD of PRAF1/RLD1 can catalyze guanine exchange on Rab8a, one of RAB-E subclass members^36^. Previous studies have suggested that RAB-E subclass members act in post-Golgi trafficking to the PM^37,38^ together with the exocyst complex^39^. This raises the possibility that RLD might act as a guanine exchange factor (GEF) for RAB-E in the exocytic pathway involving PIN trafficking. Although further investigation of the relationship among RAB-E, exocysts, and RLD is required, the idea is consistent with the *gnom*-like phenotype of the *rld* quadruple mutant embryo and with the BFA-sensitive phenotype of *rld1 rld4* double-mutant roots (Fig. 1 and Supplementary Fig. 7). Considering the expression patterns of *RLD*s and the pleiotropic phenotype of *rld* multiple mutants, RLD1 to 4 redundantly modulate auxin transport and possibly other cellular processes through the regulation of membrane trafficking in various tissues and developmental stages. Particularly in the statocytes, modulation of auxin transport by RLD1 and RLD4 is likely to be linked to gravity signaling by the LZY function. We also demonstrated that the CCL domain of LZY and the BRX domain of RLD form a complex with high affinity in vitro and that they are necessary and sufficient for the recruitment of RLD from the cytosol to the PM (Fig. 3). Both the CCL domain^13^ and the BRX domain specifically expressed in the statocytes enhanced the GSA phenotype both in the wild type and the *lzy1 lzy2 lzy3* background (Supplementary Fig. 16), possibly due to the interference of LZY-RLD interaction. These results suggest that the interaction between LZY and RLD is a key process in gravity signaling in root statocytes.

The BRX domain was initially identified as a conserved domain that often occurs as a tandem repeat in BRX and BREVIS RADIX-LIKE (BRXL) proteins^26^. Here, we have provided the first structural basis for the BRX domain as a protein–protein interaction domain that forms a 1:1 complex with LZY CCLs. The BRX domain is folded into a compact globular structure, although it is composed of only ~60 residues (Fig. 4). When the results of this structure are compared with known structures in PDB by the DALI server^40^, a module similar to the BRX domain (β-β-β-α-α-fold) often occurs as part of a larger fold in which extra α-helices or β-strands pack to support the folding of the BRX domain-like module, while they share low sequence similarity with the BRX domain (<10%). Therefore, the BRX domain is a novel domain with a specific structure and function. Our gel filtration analysis showed that the isolated BRX domains of RLDs were eluted later than the complexes with LZY CCLs, suggesting that the BRX domains of RLDs exist as monomers. However, they tend to aggregate and precipitate at higher concentrations (>500 μM), presumably due to the non-specific interactions mediated by the hydrophobic surface between the β3-strand and the α1-helix responsible for the interaction with LZYs. This is consistent with the notion that the binding to LZY3 CCL that covers the hydrophobic surface significantly improves the solubility of the RLD2 BRX domain (>10-fold increase). In contrast to the RLDs, the BRX domain of BRX is suggested to serve as a protein–protein interaction domain mediated by homotypic and heterotypic interactions. This difference might reflect the different properties of the hydrophobic groove in the β3-strand and the α1-helix, otherwise a tandem repeat might be required for the oligomerization of the BRX domain. Notably, the sequence alignment of the BRX domains based on structure shows high conservation of the residues interacting with LZYs; among 13 residues responsible for LZY binding, 9 and 12 residues are conserved in the N-terminal and C-terminal of the tandem BRX domains in BRX/BRXL, respectively.

Polar auxin transport is strictly controlled and maintained during development such as axis formation and organogenesis, while it changes rapidly in response to the environmental cue during gravitropism. Here, we demonstrated the polar localization of LZY3, which facilitates asymmetric auxin distribution toward the lower flank of the root^13^, to the PM of the lower side of the columella cell in stage 2 LRs. Our results strongly suggest that the PM is the site of action of the LZY proteins. On the other hand, the mode of binding of LZY to the PM is still unknown. LZY might bind to the PM by associating with other membrane proteins or by direct interaction with membrane lipids. There are proteins that bind reversibly to membrane lipids through electrostatic interaction^41,42^. Proteins harboring a cluster of positively charged amino acids can bind to negatively charged anionic phospholipids^43,44^. Interestingly, there are K/R-rich regions in LZY proteins that possibly contribute to membrane binding. Further investigations on the mode of membrane binding are important for understanding the regulatory mechanism of LZY polarization upon gravistimulation. Amyloplast relocation and repolarization of LZY3 occurred 30 min after gravistimulation in the stage 2 LRs (Fig. 6). Following the repolarization of LZY3, a relatively higher fluorescent signal of RLD1-GFP became obvious and was polarized in the columella cell toward the direction of gravity. Although the amount of LZY3 protein might be quite low in the cell, LZY3 could capture RLD1 around the PM due to the high affinity between the CCL and BRX domains. It has been reported that PIN3 localization is polarized in the PM of the columella cells in response to gravistimulation^14,15^. Although we did not observe polar localization of PIN3-GFP in a columella cell, a shift in the asymmetry of PIN3-GFP toward the direction of gravity in lateral columella cells was observed 300 min after gravistimulation (Fig. 6). Our results prompted us to propose a model in which LZY3 recruits RLD1 to the PM of the columella cell in a polarized manner according to the direction of gravity following amyloplast sedimentation. Subsequently, RLD might regulate PIN3 trafficking as discussed above, leading to asymmetric auxin flow (Fig. 7j). Consistently, PIN1- and PIN3-GFP signal intensities are decreased in the embryo or in columella cells of *rld* multiple mutants, respectively, and a decrease in PIN3-GFP asymmetry is also observed in *rld1 rld4*. It has also been reported that polar targeting to the PM and the efflux activity of PIN proteins are regulated by phosphorylation mediated by members of the Arabidopsis AGCVIII Ser/Thr protein kinase family^45,46^. A member of the D6 PROTEIN KINASEs, PROTEIN KINASE ASSOCIATED WITH BRX (PAX), is involved in protophloem sieve element differentiation together with BRX with double BRX domains. Considering that neither PAX nor BRX affect the PIN1 abundance or localization^46^, the mode of action of BRX in regulating auxin transport is likely to be distinct from that of RLD, implying that each BRX domain might interact with a distinct partner to execute its function. Our structural data on the BRX domain of RLD could provide valuable information to help us understand the function of the BRX family proteins.

## Methods

### Plant materials and growing conditions

In the present study, *A. thaliana* accession Columbia-0 (Col) was used as the wild-type line. The following mutant alleles and marker lines were used: *lzy1 lzy2 lzy3*^13^, *DR5rev:GFP*^28^, *PIN3-GFP*^16^, *ADF9p:GUS*^13^, and *LZY3p:LZY3-mCherry*^13^. *rld1-1* (SALK_138887C), *rld1-2* (SALK_067605C), *rld2-1* (SALK_042345C), *rld3-1* (SALK_059345), *rld3-2* (SAIL_503H11), and *rld4-2* (SALK_072865C) were obtained from the Arabidopsis Biological Resource Center^47,48^, whereas *rld2-2* (GABI_822A03) and *rld4-1* (GABI_225B01) were obtained from GABI-Kat^49^. All the T-DNA lines were backcrossed with Col at least three times prior to the construction of multiple mutants and phenotype analysis. Surface-sterilized seeds were sown on MS plates [1× Murashige Skoog salts, 1% (w/v) sucrose, 0.01% (w/v) myoinositol, 0.05% (w/v) MES (2-(*N*-morpholino) ethanesulfonic acid), and 0.5% (w/v) gellan gum; pH 5.8], incubated in the dark at 4 °C for 2–3 days, grown at 23 °C in a growth chamber under continuous light for 10–14 days, transplanted to soil, and grown under continuous light.

### Yeast two-hybrid screening and interaction assay

Matchmaker^TM^ Gold Yeast Two-Hybrid System Kit (Clontech) was used for library screening and interaction assay. The cording region for LZY3 was fused to the C terminus of GAL4 DNA-binding domain (DBD) of pGBKT7, and then the plasmid was transformed into Y2HGold yeast strain (Clontech) using Fast^TM^-Yeast Transformation Kit (G-Biosciences). Library screening was performed with Mate&Plate^TM^ Librar-Universal Arabidopsis (Normalized) (Clontech) according to the manufacturer’s instruction. Positive clones with blue color that grew on -Leu/-Trp media containing X-α-Gal (40 μg/ml) and aureobasidin A (125 ng/ml) were picked and streaked on -Leu/-Trp/-His/-Ade media containing X-α-Gal and aureobasidin A. Then colonies with blue color were selected and the plasmids were prepared from the colonies and sequenced. The same genes included in multiple independent yeast colonies were selected as candidate genes for LZY3-interacting proteins (Supplementary Table 1).

For interaction assay, full length or portion of LZY or RLD was fused to the C terminus of GAL4 DBD of pGBKT7 or GAL4 activation domain (AD), and then the plasmids were transformed into Y2HGold or Y187, respectively. It was confirmed that all constructs do not have auto-activation capability. After mating, spot assays were performed on -Leu/-Trp/-His/-Ade media by incubating for 2 days at 30 °C.

### IP and liquid chromatography with tandem mass spectrometry analysis

Two-week-old seedlings of transgenic plants carrying *35S:GFP*, *35S:LZY2-GFP*, or *35S:LZY3-GFP* (0.5 mg) were homogenized with an extraction buffer [50 mM Tris-HCl (pH 8.0), 150 mM NaCl, 1 mM EDTA, 1% (v/v) Triton X-100, cOmplete Mini Protease Inhibitor Cocktail (Roche)]. After incubation on ice for 20 min, the extract was centrifuged at 2500 × *g* for 20 min. The supernatants were centrifuged at 20,000 × *g* for 20 min and then the supernatants were used following IP. IP analyses were performed with the μMACS Epitope-Tagged Protein Isolation Kit (Miltenyi Biotec) according to the manufacture’s instruction with small modifications. The supernatants were incubated with μMACS anti-GFP microbeads for 2 h at 4 °C on a rotator and then applied to μMACS column. After washing the column, the proteins were eluted with sodium dodecyl sulfate-polyacrylamide gel electrophoresis (SDS-PAGE) sample buffer. The IP products were separated using a ready-made 12.5% (w/v) SDS-polyacrylamide gel (DRC) and stained with Flamingo (Bio-Rad). Each lane was sliced into four bands of equal length. Each gel band was washed twice with high-performance liquid chromatography (HPLC)-grade water containing 60% (v/v) acetonitrile (Kanto Chemical)/50 mM ammonium bicarbonate. Next, the gel was incubated in 10 mM dithiothreitol/50 mM ammonium bicarbonate for 45 min at 56 °C, followed by 55 mM iodoacetamide/50 mM ammonium bicarbonate for 30 min at room temperature. The incubated gel was washed twice with HPLC-grade water containing 60% (v/v) acetonitrile/50 mM ammonium bicarbonate and dried in a vacuum concentrator. The dried gel pieces were treated with 2 µl of 10 ng µl^–1^ trypsin (MS grade gold; Promega)/50 mM ammonium bicarbonate and incubated at 37 °C for 16 h. The digested peptides were recovered to a new tube. The gel was treated twice with 20 µl of 0.2% (v/v) formic acid (Wako)/50% (v/v) acetonitrile, and then all extracted peptides were collected into a tube. The extracts were dried in a vacuum concentrator and dissolved in 0.1% (v/v) formic acid/5% (v/v) acetonitrile. The dissolved solution was filtered by the Ultrafree*-*MC Centrifugal Filters (PVDF 0.45 µm; Millipore) to avoid contamination of gel pieces. Then, the peptide solution was analyzed. Liquid chromatography with tandem mass spectrometry analysis was performed by using a HTC-PAL/Paradigm MS4 system coupled to a LTQ-Orbitrap XL (Thermo Fisher Scientific) mass spectrometer. The spectra obtained were compared with a protein database (TAIR10) using the MASCOT server (version 2.4). The mascot search parameters were as follows: set off the threshold at peptide tolerance at ±10 p.p.m. and fragment mass tolerance at ±0.5 Da. Hit proteins were compared between *35S:LZY2-GFP* and *35S:GFP* or *35S:LZY3-GFP* and *35S:GFP*, and then proteins included in both LZY-GFP and GFP were excluded from the candidates for interactors. Then, the proteins co-immunoprecipateted both with LZY2-GFP and LZY3-GFP were listed as candidates for LZY interactors (Supplementary Table 2).

### The analysis of LR growth angle

For the analysis of mature LRs, the seedlings were vertically grown on MS plates for 12 days. Photographs were taken, and the angle between the direction of gravity and LR tip growth was measured using the Image J software. For the analysis of LRs in the *LZY3-mCherry*-inducible system, the seedlings were vertically grown on MS medium for 4 days, transferred to new MS medium with or without 1 μM estradiol (10 mM stock in EtOH), and incubated vertically at 23 °C under continuous light for additional 6 days. Control treatments contained an equivalent amount of solvent. Photographs were taken, and the LR length and the angle between the horizontal direction and LR tip growth were measured using the Image J software.

### GFP/mCherry imaging in columella cells of LRs

PR and LR samples were fixed in 4% (w/v) paraformaldehyde in MTSB (15.1 g/l PIPES, 1.23 g/l MgSO_4_·7H_2_O, and 1.9 g/l EGTA; pH 7.0) for more than 30 min keeping the samples in vertical orientation. After washing twice with MTSB for 1 min, respectively, fixed samples were cleared with ClearSee solution for more than 4 days^32^. Confocal images of GFP and mCherry fluorescence were obtained with FV1000 (Olympus), LSM780 (Zeiss), and TCS SP8 (Leica). We classified LR development into tripartite stages according to development of columella cells as described in Taniguchi et al.^13^ and Kiss et al.^50^. Stage 1 LRs correspond to type 2 roots harboring two rows of columella cells^50^. Stage 2 LRs correspond to types 3 and 4 with elongating columella cells, and stage 3 corresponds to type 5 with fully elongated columella cells. Stage 3 may correspond to LR stage II of the classification by Rosquete et al.^25^.

### GUS staining

Roots were incubated in GUS staining solution [100 mM sodium phosphate (pH 7.0), 10 mM EDTA, 10 mM ferricyanide, 10 mM ferrocyanide, 0.1% Triton X-100, and 2 mM 5-bromo-4-chloro-3-indolyl-β-d-glucuronic acid] at 37 °C. For whole-mount observation, samples were rinsed with 70% ethanol and cleared in chloral hydrate solution (8 g chloral hydrate, 1 ml glycerol, and 2 ml water). Samples were observed under a light microscope (BX52, Olympus) equipped with a CCD camera (DP73, Olympus).

### Transient assay with protoplasts

Plasmids and carrier DNA were introduced into protoplasts generated from Arabidopsis suspension culture in buffer containing 0.4 M mannitol and 32% (w/v) polyethylene glycol (PEG) 6000^51^. Confocal images of GFP and mCherry fluorescence were obtained with FV1000 (Olympus).

### Protein expression and purification

DNA fragments were amplified by the polymerase chain reaction and cloned into the pET47-b [+] vector (Merck Millipore) or pGEX vector (GE Healthcare). All plasmids were verified by DNA sequencing and transformed into *Escherichia coli* strain BL21Star (DE3) (Invitrogen) cells for protein expression. RLD2 (residues 1006–1066) and LZY3 (residue 274–287) were cloned into pET47b vector and pGEX vector, respectively. The RLD2 protein was expressed at 20 °C in Luria–Bertani medium containing 0.1 mM isopropyl-β-d-thiogalactopyranoside (IPTG) for 16–24 h. Harvested cells were suspended in buffer A (20 mM Tris-HCl buffer (pH 8.0) containing 150 mM NaCl) and then disrupted by sonication using Q500 sonicator (Qsonica) with 70% output intensity on ice for 30 min. After sonication, lysates were centrifuged using Optima XE-90 (Beckman Coulter) at 35,000 r.p.m. for 50 min at 4 °C and the supernatant was applied onto a cOmplete His-Tag Purification Resin (Roche). After washing with buffer A containing 20 mM imidazole, proteins were eluted with buffer A containing 250 mM imidazole. Eluted protein was treated with HRV3C protease for ~10 h at 4 °C to remove the N-terminal hexahistidine tag and purified by gel filtration (Superdex 75 pg, GE Healthcare) chromatography in buffer A. The LZY3 was expressed at 37 °C in Luria–Bertani medium containing 0.5 mM IPTG for 4–5 h. Harvested cells were suspended in buffer A, disrupted by sonication and centrifuged. After centrifugation, the supernatant was applied onto a glutathione sepharose 4B resin (GE Healthcare). After washing with buffer A, proteins were eluted with buffer A containing 20 mM glutathione. Eluted protein was treated with HRV3C protease for ~10 h at 4 °C to remove the N-terminal GST tag and purified by gel filtration (Superdex 75 pg, GE Healthcare) chromatography in buffer A. The proteins were further purified by reverse phase chromatography (Resource RPC, GE Healthcare) with a linear gradient of 10–90% acetonitrile. The eluted proteins were collected and the solvent was evaporated by a vacuum concentrator. The yielded peptide powders were stored at −30 °C until use.

For crystallization of the RLD2-LZY3 protein complex, RLD2 (residues 1006–1066) and LZY3 (274–287) were co-expressed at 20 °C in Luria–Bertani medium containing 0.1 mM IPTG for 16–24 h. Harvested cells were suspended in buffer A, disrupted by sonication, and centrifuged. After centrifugation, the supernatant was applied onto a glutathione sepharose 4B resin (GE Healthcare). After washing, proteins were eluted and treated with HRV3C protease for ~10 h at 4 °C. The proteins were purified by gel filtration (Superdex 75 pg, GE Healthcare) chromatography in buffer A. RLD2 and LZY3 were co-purified under the purification described above. For structure determination, V1057M mutation was introduced into RLD2 and then selenomethione (SeMet)-labeled RLD2 and LZY3 were prepared in M9 medium containing SeMet under conditions inhibiting the methionine biosynthesis pathway^52^. The expression conditions and purification procedures were the same as those used for the native protein.

### Crystallization and data collection

Initial crystallization screening was performed using the Mosquito crystallization robot (TTP Labtech) with the commercial crystallization solution kits JCSG Core Suite I–IV and PACT Suite (Qiagen). The best crystals of the complex between RLD2 and LZY3 were obtained from solutions containing 2.5–3.0 mM of the protein complex and a reservoir solution containing Tris-HCl buffer (pH 7.0), 0.1 M sodium acetate, and 7–10% PEG 4000 at 20 °C. The best crystals of the complex between Se-Met-labeled RLD2 V1057M bound to LZY3 were obtained from solutions containing 1.5–2.5 mM of the protein complex and a reservoir solution containing 0.05 M sodium citrate buffer (pH 6.5), 0.1 M sodium acetate, and 10–15% PEG 4000 at 20 °C. The crystals were transferred stepwise into a cryoprotective solution containing 10% ethylene glycol for RLD2-LZY3 crystals and flash cooled at 100 K. X-ray diffraction data were collected at a wavelength of 1.000 Å (for native crystal) or 0.9658 Å (for Se-Met crystal) on BL41XU, and BL44XU beamlines at SPring-8 or BL-1A beamline at the Photon Factory. All data were processed and scaled using HKL-2000^53^. The crystal data are summarized in Supplementary Table 3.

### Structure determination and refinement

Phases of the Se-Met-labeled RLD2 V1057M-LZY3 complex crystal were calculated by a single-wavelength anomalous dispersion method using data collected at the peak wavelength of selenium. Selenium positions were located using the program SOLVE/RESOLVE^54^. Two RLD2 V1057M-LZY3 complexes were present in the asymmetric unit of the crystal. The built model was refined through alternating cycles using the Coot^55^ and PHENIX^56^ programs. The structure of the binary complex of RLD2-LZY3 was determined by molecular replacement using the structure of RLD2 V1057M-LZY3 complex as a starting model. Molecular replacement was performed with Phaser^57^. Model building and refinement were performed as well as those for the RLD2 V1057M-LZY3 complex structure. The refinement statistics are summarized in Supplementary Table 3. Coordinates and structure factors have been deposited in the Protein Data Bank under accession codes 6L0W (the Se-Met-labeled RLD2 V1057M-LZY3 complex) and 6L0V (the RLD2-LZY3 complex).

### Structure and sequence comparison

Multiple sequence alignments of the BRX domains and LZY family proteins were performed using CLUSTALW^58^. Pairwise structural comparisons were performed using C_α_-atom positions by the PDBeFOLD server^59^ and structure figures were prepared using the PyMOL Molecular Graphics System, Version 1.7 Schrödinger, LLC. Electrostatic potentials were calculated with APBS^60^ and are displayed in PyMol.

### Binding study by ITC analysis

ITC was conducted using a calorimeter (iTC200, GE Healthcare) at 20 °C. Purified protein samples were dialyzed overnight in buffer containing 20 mM Tris-HCl (pH 8.0) and 150 mM NaCl. We performed data fitting with a 1:1 binding model using the ORIGIN^TM^ software program supplied with the instrument. The ITC profile for binding of the RLD2 BRX domain to the LZY3 CCL was obtained by injections of 1 μl of 200 μM LZY3 (residues 274–287) into the RLD2 BRX domain solution (20 μM) at 20 °C. Raw data for 40 sequential injections and the plot of the heat evolved (kcal) per mole of LZY3 CCL added, corrected for the heat of LZY3 CCL by dilution, against the molar ratio of LZY3 CCL to the RLD2 BRX domain.

### Pull-down binding assay

All mutations were produced by site-directed mutagenesis. For in vitro pull-down binding assays, the purified protein and GST-fusion protein were mixed with a slurry of glutathione sepharose 4B and incubated at 4 °C. After washing with incubation buffer, collected eluates were subjected to SDS-PAGE. The relative amount of the proteins pulled down was measured with error bars representing standard deviation from three independent measurements.

### Gravity stimulation analysis

The seedlings were vertically grown on MS plates for 8 days, and then rotated 180° and incubated for additional 5, 30, and 60 min, respectively. The LR sample was cut out with gellan gum block and placed into a 1.5 ml tube, keeping the sample in vertical orientation. Samples were fixed in 4% (w/v) paraformaldehyde in MTSB (15.1 g/l PIPES, 1.23 g/l MgSO_4_·7H_2_O, and 1.9 g/l EGTA; pH 7.0) at room temperature for more than 30 min, keeping the samples in vertical orientation. After fixation, samples were washed twice with MTSB buffer for 1 min and cleared with ClearSee solution at room temperature for more than 4 days^32^. For observation of LZY3-mCherry, samples were mounted in ClearSee solution, and a confocal image of LZY3-mCherry was obtained using a LSM780 (Zeiss) equipped with a Plan-Apochromat ×40 oil-immersion objective with a numerical aperture of 1.4 (Zeiss). Spectral unmixing and processing of images were conducted using ZEN2012 software (Zeiss). For quantitative analysis of LZY3-mCherry localization, LZY3-mCherry fluorescence images were subjected to analysis. Central columella cells and lateral columella cells, which are situated at the second-outermost layer of the LR tip, were analyzed. The PM across the tip side of the cell was selected as a region of interest (ROI), and LZY3-mCherry fluorescence intensity was measured using the ImageJ software. ROI was equally segmented into four compartments from the basal side to the apical side, giving rise to ROI-1 (most basal), ROI-2 (second basal), ROI-3 (second apical), and ROI-4 (most apical). The ROI-1/ROI-4 ratio of LZY3-mCherry fluorescence intensity was calculated as the basal/apical ratio (Supplementary Fig. 19). For comparison of LZY3-mCherry localization, differences in the basal/apical ratio of LZY3-mCherry fluorescence intensity between before rotation, 5 min after rotation, 30 min after rotation, and 60 min after rotation were tested using the Tukey–Kramer method with a significance threshold of *P* < 0.05. For observation of RLD1-GFP or PIN3-GFP, samples were mounted in ClearSee solution, and a confocal image of RLD1-GFP or PIN3-GFP was obtained using a TCS SP8 DLS (Leica) equipped with a Plan-Apochromat ×63 oil-immersion objective with a numerical aperture of 1.4 (Leica). For quantitative analysis of PIN3-GFP localization in LR tips, lateral columella cells adjacent to central columella cells, which are situated at the second outermost layer of the LR tip, were analyzed. The fluorescence intensity of PIN3-GFP at the outer lateral domains of the PM was measured using the ImageJ software. The ratio of GFP fluorescence intensity at the apical flanks to that at the basal flanks was calculated as the basal/apical ratio (Fig. 6r).

### Starch staining

The fragments of PR containing LRs whose length was <2.5 mm were cut out from 8-day-old seedlings. Samples were fixed in 4% (w/v) paraformaldehyde in MTSB for more than 30 min with the direction of gravity. After washing twice with MTSB for 1 min respectively, fixed samples were cleared with ClearSee solution for more than 4 days^32^. Then, cleared samples were transferred to 10% (w/v) xylitol, 25% (w/v) urea, and 2.1% (w/v) sodium chloride for over 1 min, and were stained in 400 μM iodine solution (Wako), 10% xylitol, 25% urea, and 2.1% sodium chloride. This staining method was modified from recently reported one^61^.

### Quantitative and semi-quantitative RT-PCR

For real-time quantitative reverse transcription PCR (qRT-PCR), the seedlings, vertically grown on MS medium for 7 days, transferred to new MS medium with or without 1 μM estradiol (10 mM stock in EtOH), incubated vertically at 23 °C under continuous light for additional 3 days, were used. Total RNA was extracted with an RNeasy Plant Mini Kit (Qiagen). Complementary DNA (cDNA) was synthesized from 0.5 mg of total RNA treated by ReverTra Ace qPCR RT Master Mix with gDNA Remover (Toyobo) according to the manufacturer’s instructions. KAPA SYBER FAST qPCR Kit was used for the preparation of real-time qPCR mix, and then real-time qPCR was performed using the LightCycler 96 Real-Time PCR System. Based on the results of three technical repeats for three biological replicates, mesenger RNA relative expression levels (in arbitrary units) were determined using standard curves for *LZY3-mCherry* and *ACT8* generated by serial dilutions of cDNA. For semi-quantitative RT-PCR, cDNA was synthesized from 1 µg of total RNA from 10-day-old seedlings with ReverTra Ace qPCR RT Master Mix with gDNA Remover (Toyobo) according to the manufacturer’s instructions. *ACT8* was used as an internal control. All primer sequences are listed in the Supplementary Table 4.

### Plasmid construction

We used the Gateway Cloning System (Invitrogen) to construct *RLD1p:GUS*, *RLD2p:GUS*, *RLD3p:GUS*, *RLD4p:GUS*, *RLD1p:RLD1-GFP*, *ADF9p:RLD1*, *ATHB8p:RLD1-mCherry*, and *35S:RLD1-GFP*. The 2866-, 2178-, 1016-, and 2419-bp upstream from the start codon of *RLD1*, *RLD2*, *RLD3*, and *RLD4*, respectively, were used as the promoter regions and fused with DNA fragments containing *GUS* gene and *NOS* terminator on the *pENTR* vector. Subsequently, they were introduced into *pFAST-R01*^62^. The 3122- and 1700-bp fragments upstream from the start codon of *ADF9* and *ATHB8* were used as *ADF9* and *ATHB8* promoters^13,63^, respectively. The promoter regions of *RLD1*, *ADF9*, and *ATHB8* were combined with cloning sites and *NOS* terminator in *pENTR* vector. Full-length cDNA of *RLD1*, *RLD1-GFP/mCherry* fusion gene, *BRXd* of *RLD2* [3′ end of *RLD2* CDS (219 bp)] were cloned between the promoter and *NOS* terminator, followed by introducing into *pGWB501*^64^ (*RLD1p:RLD1-GFP*, *ADF9p:RLD1*, *ATHB8p:RLD1-mCherry*, *ADF9p:BRXd (RLD2)-GFP*). Full length cDNA of *RLD1* was cloned into *pENTR* vector, followed by introducing into *pFAST-R02*^62^ (*35S:RLD1-GFP*). To introduce mutations at K275 and L285 of LZY3 on *LZY3p:LZY3-mCherry* vector, PCR amplification using primers containing respective mutation was performed and then mutated *LZY3* CDS was replaced with wild-type CDS on *LZY3p:LZY3-mCherry pENTR* vector, followed by introducing into *pGWB501* (*LZY3p:LZY3(K275A&L285A)-mCherry*). To construct *G10-90p:XVE»LZY2-mCherry*, full-length of *LZY2* CDS fused with mCherry was inserted in the multi-cloning site of the *pER8* vector^65^. To construct *ADF9p:XVE»LZY3-mCherry*, the *G10–90* promoter region was replaced with the *ADF9* promoter region in *pER8* vector. The *LZY3-mCherry* fusion gene was inserted in the multi-cloning site of the *pER8 ADF9p* vector. With binary vectors carrying these constructions, plants were stably transformed using standard protocol for *Agrobacterium* (strain GV3101)-mediated transformation^66^. Full length of *RLD1* CDS (3309 bp), truncated *RLD1ΔPH* cDNA (2927 bp, deleted 5′ end 375 bp corresponding to PH domain), truncated *RLD1ΔBRX* cDNA (3051 bp, deleted 3′ end 258 bp corresponding to the BRX domain), and full length of *RLD2*, *RLD3*, and *RLD4* CDS were amplified by RT-PCR from the Col wild type and their fragments were fused with DNA fragments containing *GFP* gene and *NOS* terminator (*GFP-NOSt*) on the *pUC19* vector under the cauliflower mosaic virus *35S* promoter (*35S:RLD1-GFP*, *35S:RLD1ΔPH-GFP*, *35S:RLD1ΔBRX-GFP*, *35S:RLD2-GFP*, *35S:RLD3-GFP*, *35S:RLD4-GFP*). To construct *35S:RLD1ΔRCC1-GFP* and *35S:RLD1ΔFYVE-GFP*, upstream region of respective domains in *RLD1* CDS and downstream region of the domain were amplified by RT-PCR from the Col wild type, and amplified fragments were inserted between *35S* promoter and *GFP-NOSt* fusion gene on the *pUC19* vector using In-Fusion HD Cloning Kit (Clontech). 3′ End of *RLD1* CDS (258 bp), corresponding to the BRX domain, was fused with DNA fragments containing *mCherry* gene and *NOS* terminator (*mCherry-NOSt*) on the *pUC19* vector under the cauliflower mosaic virus *35S* promoter (*35S:BRXd-mCherry*). *mCherry* gene without stop codon and *LTI6b* CDS were amplified by PCR and inserted into the multi-cloning site on the *pUC19* vector using In-Fusion HD Cloning Kit (*35S:mCherry-LTI6b*). Then, *CCL* was fused with DNA fragments containing *mCherry-LTI6b* fusion gene and *NOSt* on the *pUC19* vector (*35S:mCherry-LTI6b-CCL*). In addition, we introduced mutations one by one at F1052 and W1066 of RLD1 on the *RLD1p:RLD1-GFP* vector by PCR amplification using primers containing respective mutation and then mutated *RLD1* CDS was replaced with wild-type CDS on 35*S:RLD1-GFP* vector [*35S:RLD1(F1052A&W1066A)-GFP*, *35S:RLD1(F1052R&W1066R)-GFP*]. To introduce mutations at K275 and L285 of LZY3 on *35S:LZY3-mCherry* vector, PCR amplification using primers containing respective mutation was performed and then mutated *LZY3* CDS was replaced with wild-type CDS on 35*S:LZY3-mCherry* vector [*35S:LZY3(K275A&L285A)-mCherry*].

### Reporting summary

Further information on research design is available in the Nature Research Reporting Summary linked to this article.

## Supplementary information

Supplementary Information

Reporting Summary

## Data Availability

The coordinates and structure factors for the Se-Met-labeled RLD2 V1057M-LZY3 complex and the RLD2-LZY3 complex have been deposited in the Worldwide Protein Data Bank with the accession codes 6L0W and 6L0V, respectively. The mass spectrometry proteomics data have been deposited to the ProteomeXchange Consortium via the PRIDE^67^ partner repository with the dataset identifier PXD016219. The authors declare that the data supporting the findings of this study are available within the manuscript and its supplementary files or are available from the corresponding author upon reasonable request. Raw data for underlying Figs. 1d–e, 2e, 6e, f, s, and 7i and Supplementary Figs. 7g–j, 12, 14c, 20g–j, and 22c are provided in the Source Data file.

## Source data

Source Data
