# Supplementary material for: Polar recruitment of RLD by LAZY1-like protein during gravity signaling in root branch angle control
Source: Nat Commun. 2020 Jan 3;11:76. doi: 10.1038/s41467-019-13729-7 (PMC6941992; doi:10.1038/s41467-019-13729-7)
Supplement: Supplementary file 1 — Supplementary Information [file 41467_2019_13729_MOESM1_ESM.pdf]

**Supplementary Information**

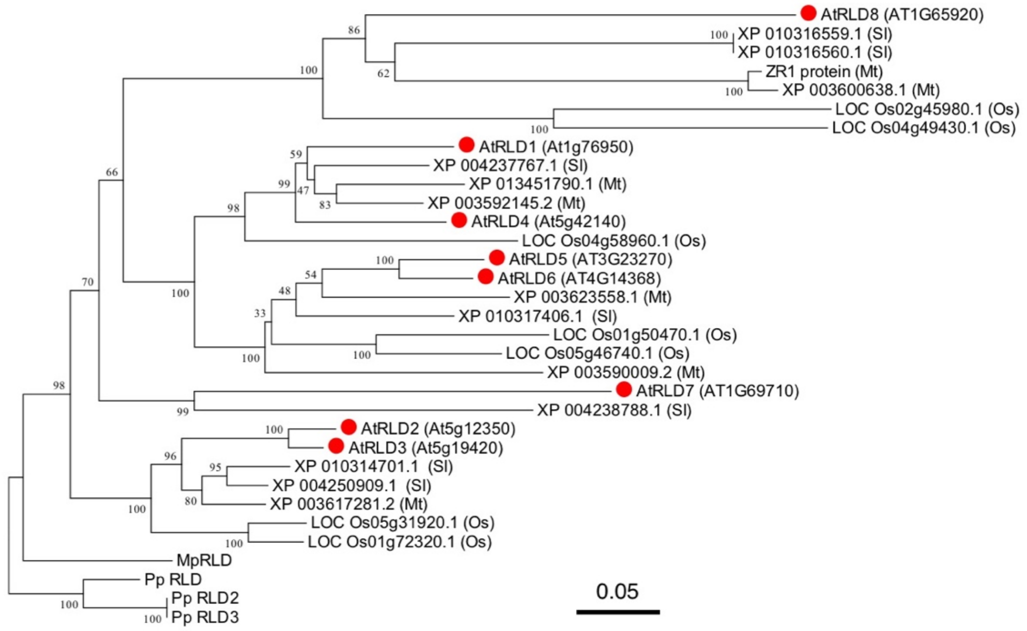


**Supplementary Fig. 1. RLD family in plants.**
The evolutionary history was inferred using the Neighbor-Joining method. The percentage of replicate trees in which the associated taxa clustered together in the bootstrap test (1,000 replicates) are shown next to the branches. Evolutionary analyses were conducted in MEGA6. Sl; *Solanum lycopersicum*, Os; *Oryza sativa*, Mt; *Medicago truncatula*. Red-filled circles show Arabidopsis RLD proteins.

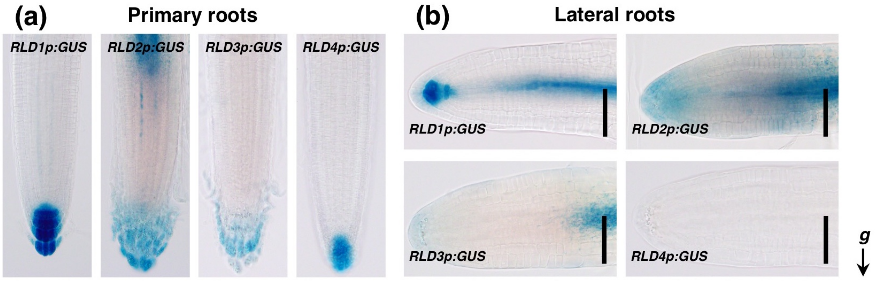


**Supplementary Figure 2. Promoter activity of *RLD* family genes in root development.**
**(a)** GUS staining of primary roots of 5-day-old seedlings harboring *RLD1p:GUS*, *RLD2p:GUS*, *RLD3p:GUS*, and *RLD4p:GUS*. **(b)** GUS staining of lateral roots of 8-day-old seedlings expressing respective *RLDp:GUS*. Arrow marked with *g* represents the direction of gravity. Scale bars, 50 μm.

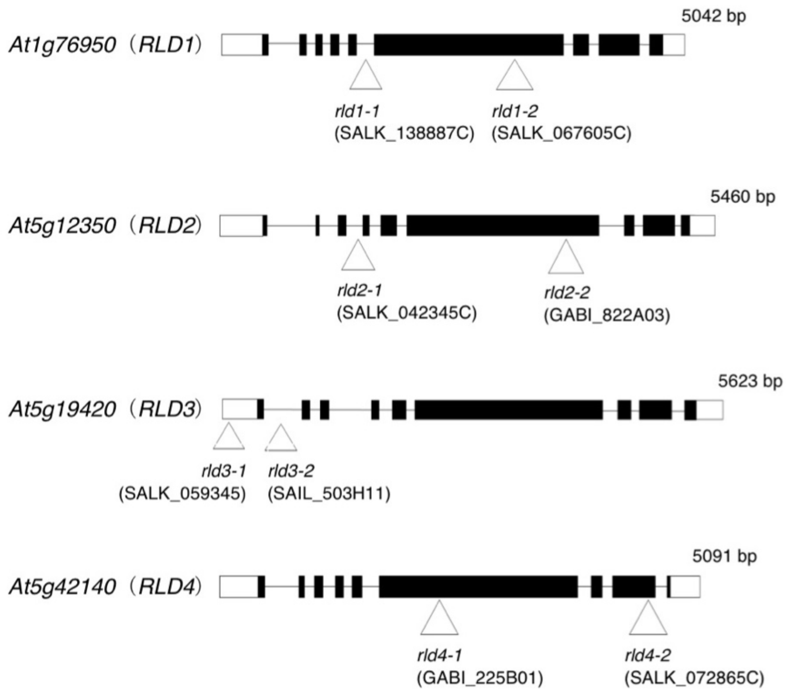


**Supplementary Fig. 3. T-DNA insertions in *RLD* family genes.**
Schematic representation of the gene structure of *RLD1*, *RLD2*, *RLD3*, and *RLD4*. Arrowhead indicates insertion position of each T-DNA in *rld1*, *rld2*, *rld3*, and *rld4*. Open boxes, filled boxes, and solid lines represent UTRs, exons, and introns, respectively. Number indicates the genomic length of each *RLD* gene from the start of 5'UTR to the end of 3'UTR.

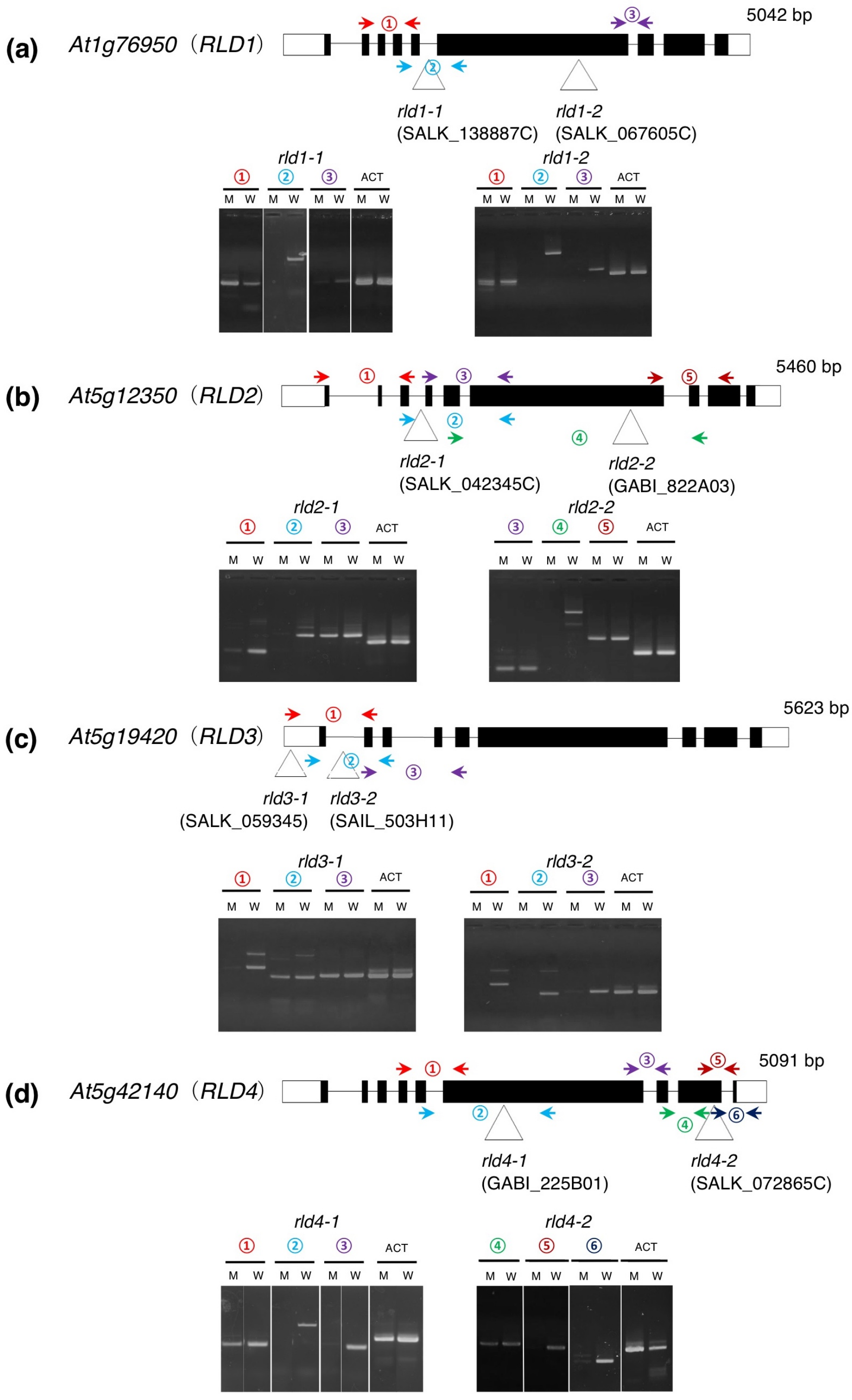


**Supplementary Fig. 4. RT-PCR in respective *rld* mutants.**
**(a–d)** Schematic representation of the gene structure of *RLD1* (a), *RLD2* (b), *RLD3* (c) and *RLD4* (d). Arrowhead indicates insertion position of each T-DNA in *rld* mutants. Arrows indicate positions of primers. Transcript levels of *RLD1* (a), *RLD2* (b), *RLD3* (c), *RLD4* (d) and *ACTIN8* (*ACT*) in 10-day-old seedlings were examined by RT-PCR with primers indicated by arrows. M; each *rld* mutant, W; wild-type Col.

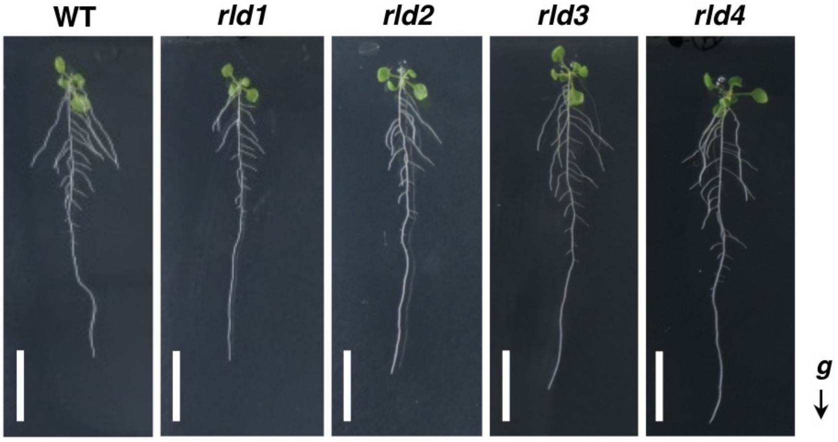


**Supplementary Fig. 5. Phenotypes of *rld* single mutants.**
10-day-old seedlings of Col, *rld1-2*, *rld2-2*, *rld3-2*, and *rld4-1*from the left. Scale bars, 2 cm.

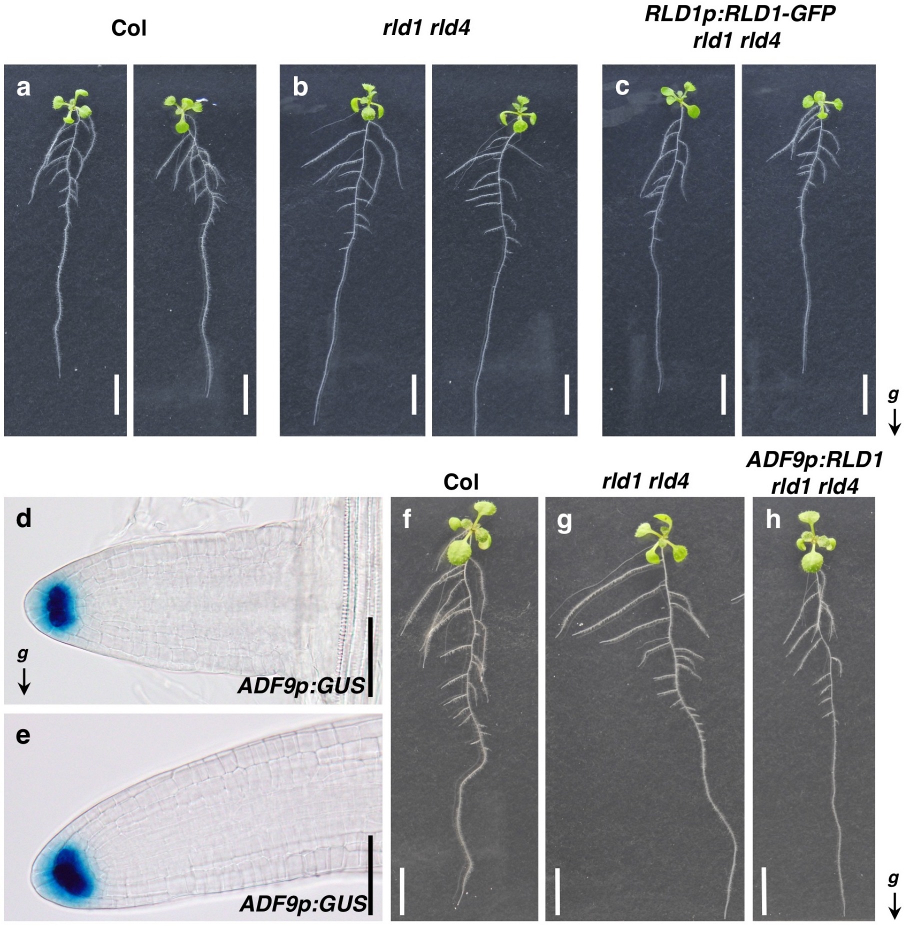


**Supplementary Fig. 6. Transformation-rescue analyses in GSA control of lateral roots. (a–c)** 12-day-old seedlings of Col (a), *rld1-2 rld4-1* (b), and *rld1-2 rld4-1* harboring *RLD1p:RLD1-GFP* (c). **(d, e)** *ADF9* promoter activity in lateral roots of 8-day-old seedlings of *ADF9p:GUS* expressing plants at the developmental stage just before (d) and after bending (e). **(f–h)** Transformation-rescue analysis with statocyte-specific *RLD1* gene. 12-day-old seedlings of Col (f), *rld1-2 rld4-1* (g), and *rld1-2 rld4-1* harboring *ADF9p:RLD1* (h). Scale bars, 50 μm (d, e) and 2 cm (a–c, f–h). Arrow marked with *g* represent the direction of gravity.

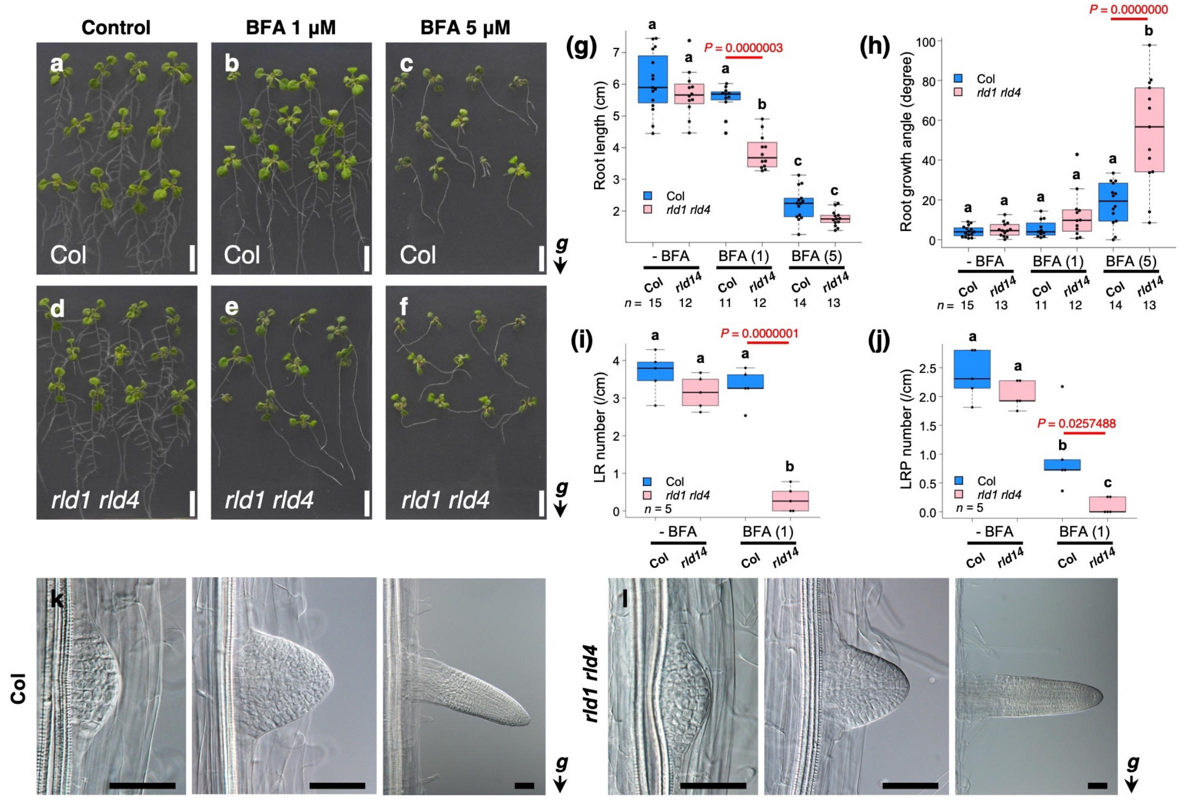


**Supplementary Fig. 7. *rld1 rld4* double mutants were more sensitive to BFA treatment.** **(a–f)** 10-day-old seedlings of Col (a–c) and *rld1-2 rld4-1* (d–f), grown on MS medium plate containing EtOH (a, d), 1µM BFA (b, e), and 5 µM BFA (c, f). Arrow marked with *g* represent the direction of gravity. **(g–j)** Box plot showing the effects of BFA treatment on primary root length (g), growth angle (h), emerged lateral root (LR) density (i), and LR primordia (LRP) density (j) in 10-day-old seedlings of Col and *rld1-2 rld4-1* double mutant. Median and quartile values are provided by the central line and box boundaries. Whiskers show min to max values. *n*, sample number of three biologically independent experiments. Different letters in each graph indicate the statistical differences (Tukey-Kramer, $P < 0.05$). **(k, l)** The LR development in 10-day-old seedlings of Col (k) and *rld1-2 rld4-1* double mutant (l) at the LRP (left), LR emergence (middle), and LR bending stage (right). Scale bars, 1 cm (a–f) and 50 µm (k, l). Source data for (g–j) are provided as a Source Data file.

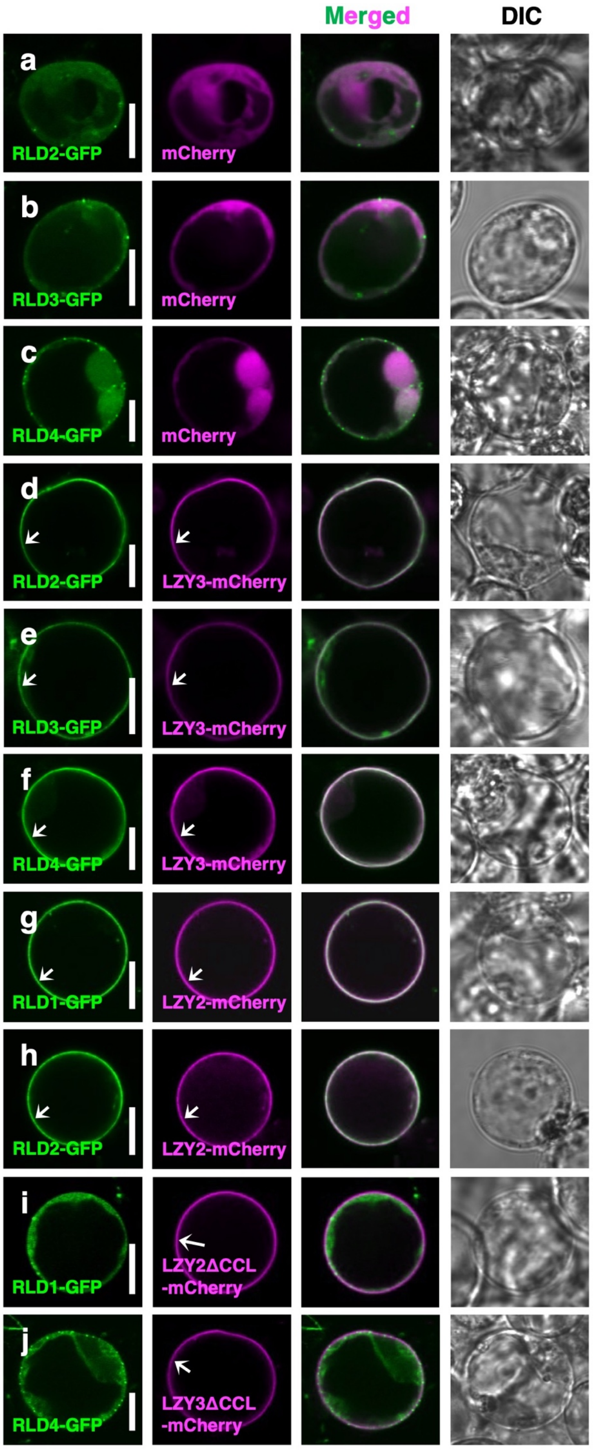


**Supplementary Fig. 8. LZY-dependent RLD localization in the plasma membrane of Arabidopsis protoplast cells.**
**(a–c)** Co-expression of RLD2-GFP (a), RLD3-GFP (b), and RLD4-GFP (c) with mCherry in Arabidopsis protoplast cells. **(d–f)** Co-expression of RLD2-GFP (d), RLD3-GFP (e), and RLD4-GFP (f) with LZY3-mCherry. Arrows indicate plasma membrane-localized signals. **(g, h)** Co-localization of RLD1-GFP (g) and RLD2-GFP (h) with LZY2-mCherry in Arabidopsis protoplast cells. **(i, j)** Co-expression of RLD1-GFP with CCL-deleted LZY2-mCherry (LZY2ΔCCL-mCherry) (i), and of RLD4-GFP with CCL-deleted LZY3-mCherry (LZY3ΔCCL-mCherry) (j) in Arabidopsis protoplast cells. Scale bars, 10 μm.

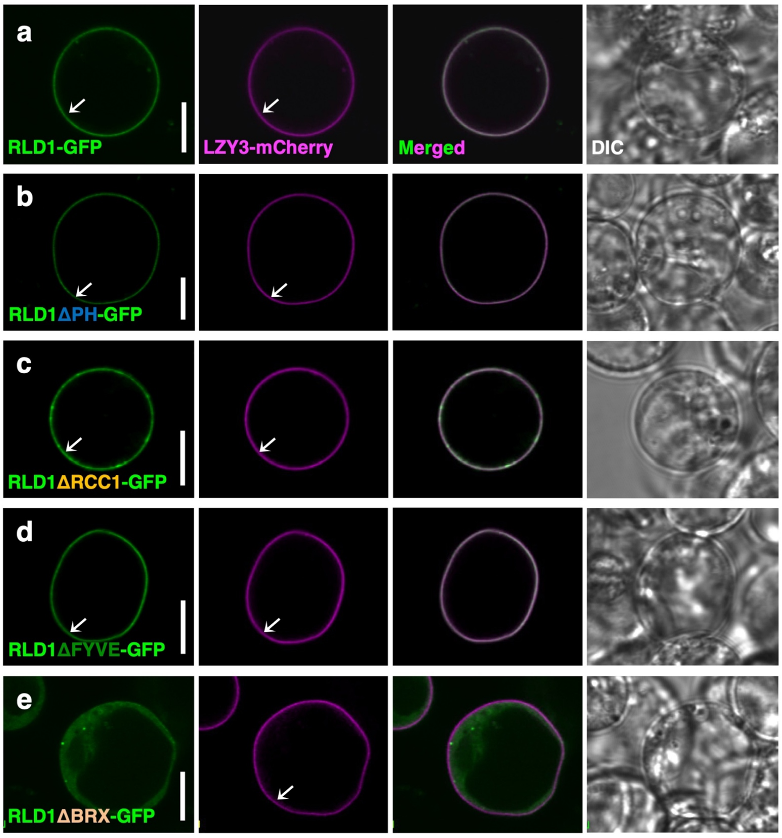


**Supplementary Fig. 9. Truncated RLD1-GFP localization in LZY3-mCherry expressing Arabidopsis protoplast cells.**

**(a–e)** Co-expression of LZY3-mCherry with RLD1-GFP (a), PH-deleted RLD1-GFP (RLD1ΔPH-GFP) (b), RCC1-deleted RLD1-GFP (RLD1ΔRCC1-GFP) (c), FYVE-deleted RLD1-GFP (RLD1ΔFYVE-GFP) (d) and BRX-deleted RLD1-GFP (RLD1ΔBRX-GFP) (e) in Arabidopsis protoplast cells. Arrows indicate plasma membrane-localized signals. Scale bars, 10 μm.

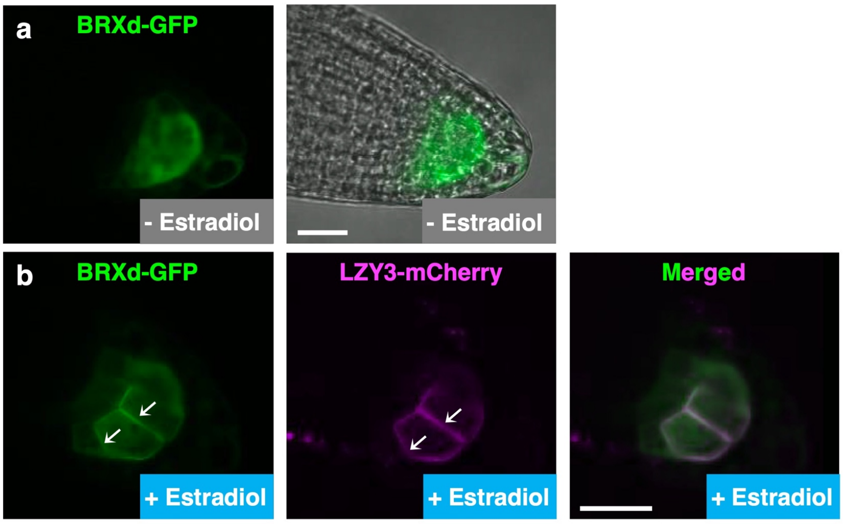


**Supplementary Fig. 10. LZY3-mCherry recruited GFP fused with BRX domain of RLD2 to the plasma membrane in columella cells.**
**(a, b)** 11-day-old seeding harboring *ADF9p:BRXd (RLD2)-GFP* & *pER8 ADF9p:LZY3-mCherry*, transferred to MS medium plate containing EtOH (a) and 1 μM estradiol (b) at 10 days old. Arrows indicate plasma membrane-localized signals. Scale bars, 20 μm.

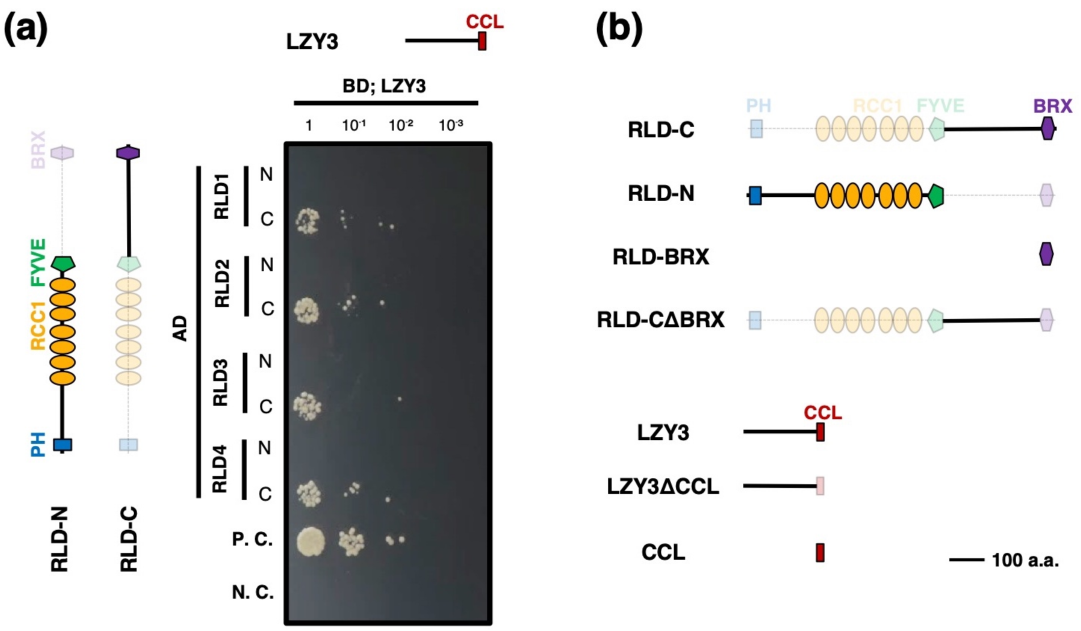


**Supplementary Fig. 11. Yeast two-hybrid assay using truncated RLD and LZY3.**
**(a)** Interaction between LZY3 and N- or C-terminus of RLDs in the Y2H system. Interaction was indicated by growth on selection medium lacking leucine, tryptophan, histidine and adenine. **(b)** Schematic diagrams of truncated forms of RLD and LZY3 used for the Y2H system in Figure 3 (j, k).

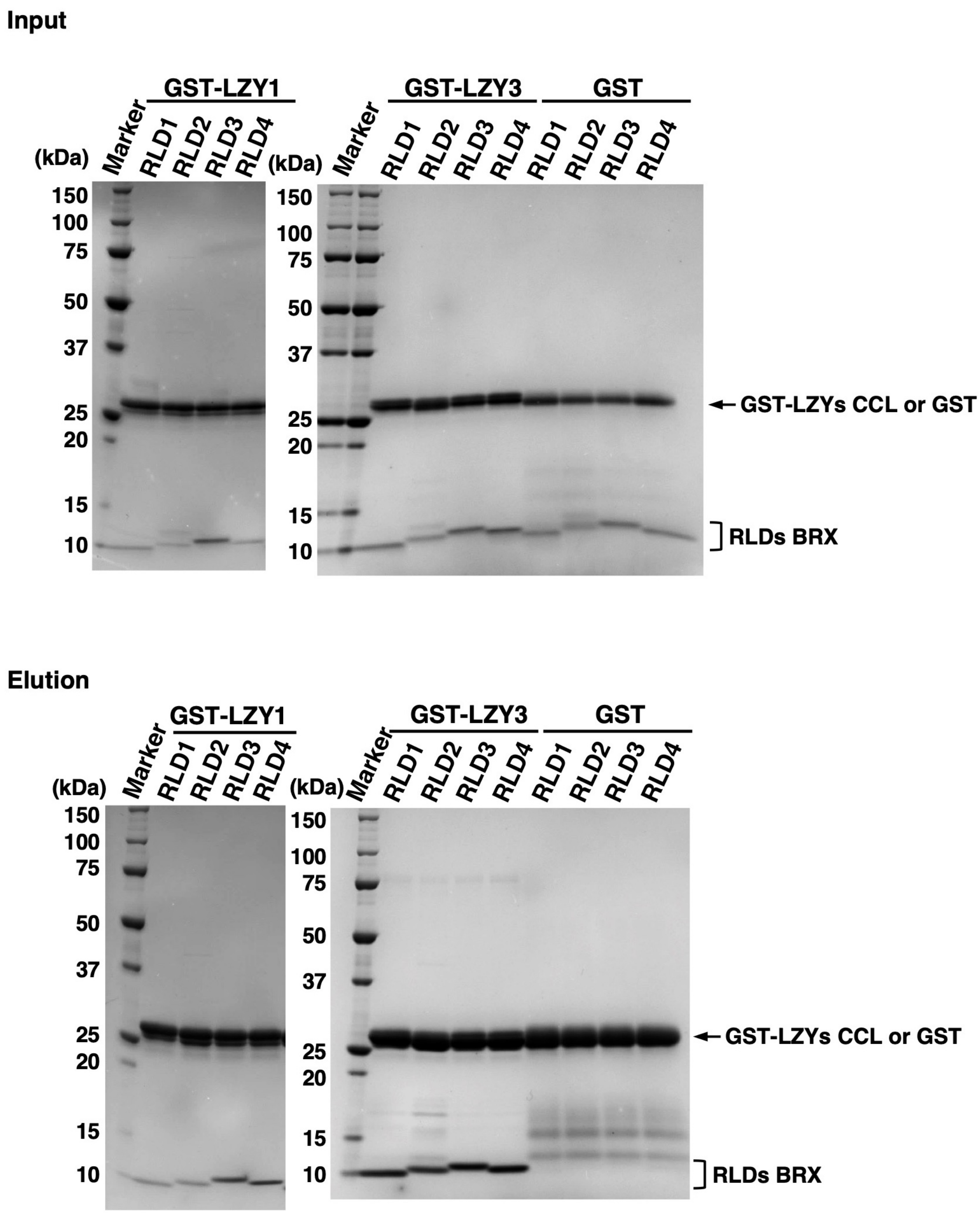


**Supplementary Fig. 12. The interaction between RLDs BRX domain and LZYs CCL.** Pull-down binding assay. RLD1-RLD4 BRX domains were pulled down with GST-LZY1 CCL or GST-LZY3 CCL and analyzed by SDS-PAGE. GST was used as a negative control. The experiments were done for more than three times at the same condition and the representative result was shown. All of the CCLs of LZY1, LZY2 and LZY3 bound to the all BRX domains of RLD1, RLD2, RLD2 and RLD4 since LZY2 CCL sequence is completely identical to LZY3 CCL (Fig. 5a). Source data are provided as a Source Data file.

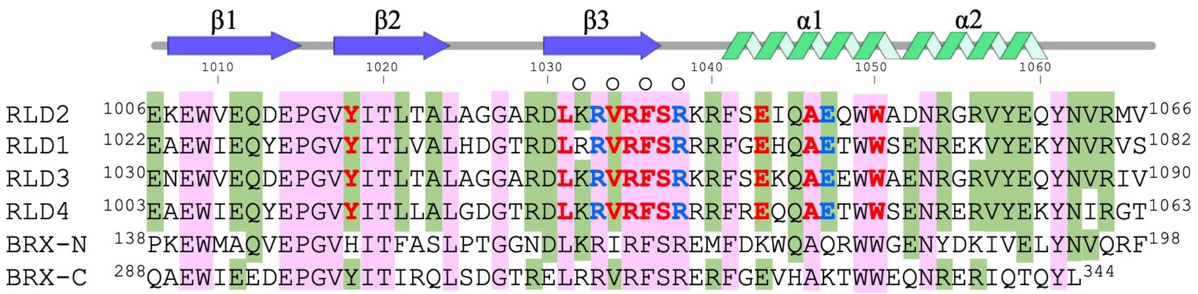


**Supplementary Fig. 13. Sequence alignments of BRX domains**

Structure-based sequence alignment of BRX domains in *Arabidopsis thaliana* RLD1–RLD4 and BRX. BRX have two BRX domains, BRX-N (residues 138–198) and BRX-C (residues 288–344). Completely conserved residues and modestly conserved (greater than 65%) are filled in magenta and green, respectively. Secondary structures of RLD2 are shown at the top with helix (spiral) and β-strand (arrow). In the interaction between RLD2 and CCL, residues whose side chain atoms form inter-molecular hydrogen bonds and residues making intermolecular Van der Waals contacts are colored in cyan and red, respectively while residues whose main chain form inter-molecular hydrogen bonds are indicated with open circle.

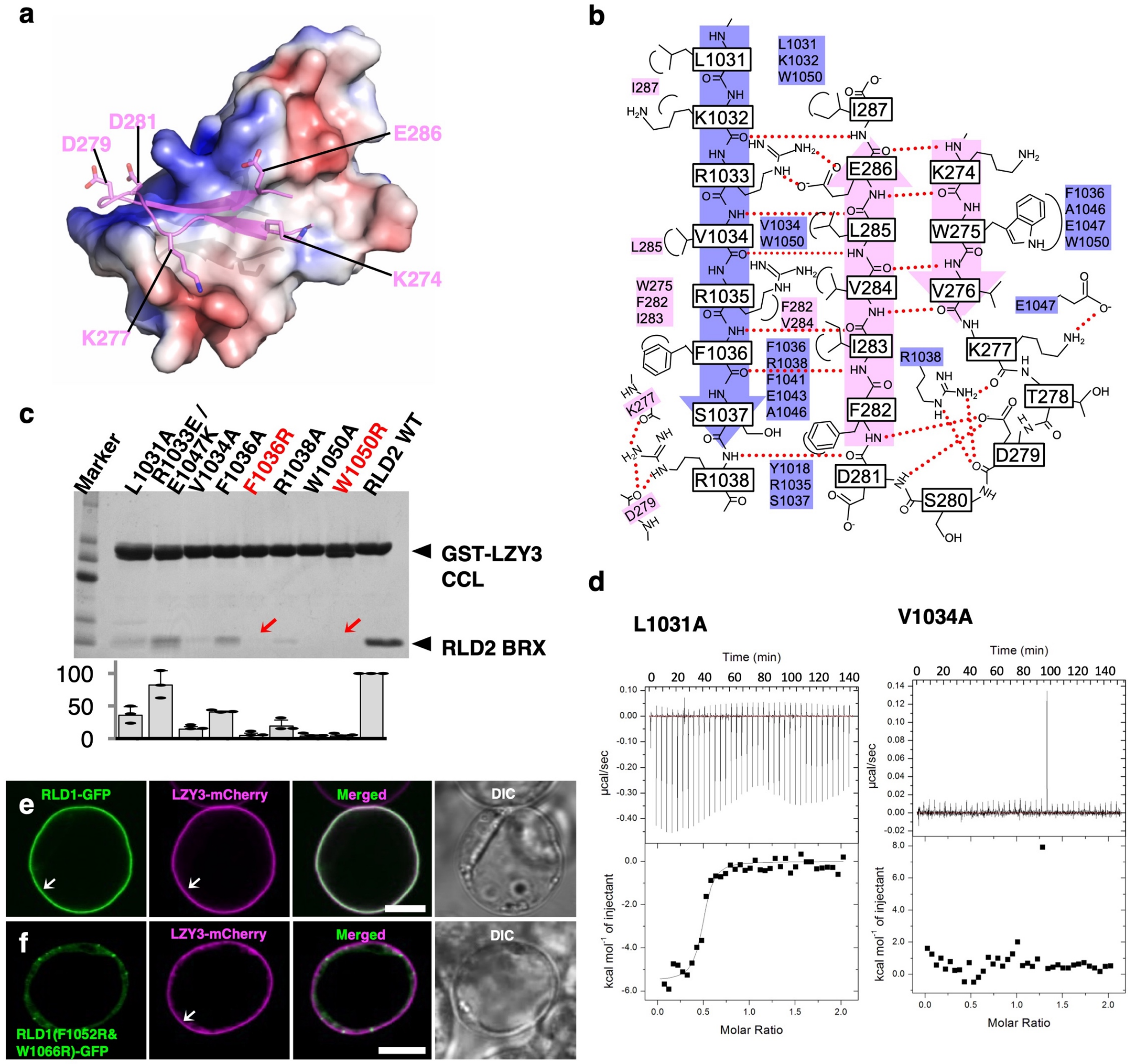


**Supplementary Fig. 14. The interaction between RLD2 BRX domain and LZY3 CCL.** **(a)** The electrostatic surface potential of RLD2 BRX domain with positive (blue) and negative (red) charges. The acidic or basic residues of LZY3 shown in stick models are electrostatically complementary to the surface of RLD2 BRX domain. **(b)** A schematic representation of the interaction between RLD2 BRX domain and LZY3 CCL. Hydrogen bonds are shown as dotted lines and van der Waals contacts are shown. **(c)** Pull-down binding assay. RLD2 BRX domains (WT and mutants) were pulled down with GST-LZY3 CCL and analyzed by SDS-PAGE (top panel) and quantification of the relative amount of pulled down RLD2 BRX domain (bottom panel). Arrows indicate the reduced abundance of RLD2 BRX (Lanes, F1036R and W1050R). Data consist of three independent samples with SD. Source data are provided as a Source Data file. **(d)** Binding of the RLD2 BRX domain mutants (L1031A, V1034A) to the LZY3 CCL peptide. The obtained ITC profile (RLD2 L1030A) resulted in the KD value of 146±46 nM, while the N = 0.45. Relatively low N value suggests that subjected protein sample was partially denatured. The obtained ITC profile (RLD2 V1034A) resulted in no significant heat change was observed. We found samples were precipitated after injections were completed and little heat change was observed when LZY3 CCL peptide was titrated into each RLD2 mutant. We tried ITC measurement using other mutants (F1036A, R1038A, W1050A), but results were

same as in V1034A mutant. RLD2 BRX domain tends to aggregate without the interaction with LZYs because of the hydrophobic nature of the LZY-binding region. RLD2 mutant might be more unstable than wild type in the ITC measurement condition which is different from that used in the pull-down binding assay in the aspect of concentration, temperature and operating time. **(e, f)** Co-expression of LZY3-mCherry with RLD1-GFP (e) and mutated RLD1-GFP (F1052R and W1066R, corresponding to F1036R and W1050R in RLD2) (f) in Arabidopsis protoplast cells. Arrows indicate plasma membrane-localized signals. Scale bars, 10 μm.

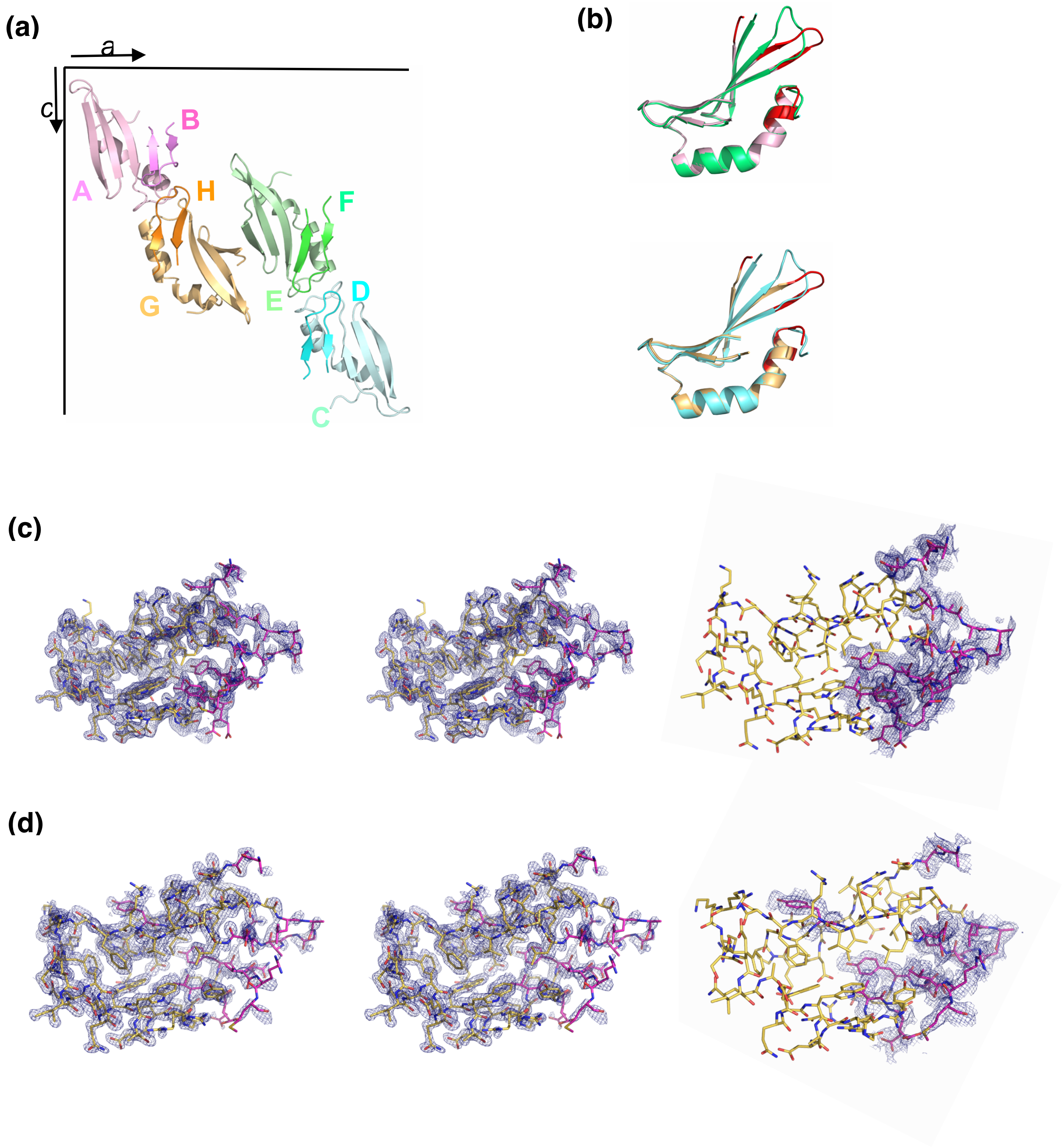


**Supplementary Fig. 15. The structure of the CCL-BRX complex.**
**(a)** The four RLD2-LZY3 complexes in the asymmetric unit are shown with their chain ID. Two of four complexes are related by pseudo-translational symmetry. The unit cell axes *a* and *c* are shown with arrows. **(b)** The BRX domains related by pseudo-translational symmetry are superimposed on each other (upper; protomer A-E, bottom; protomer C-G). Residues relatively lower fitting to electron density (high real-space R-value Z-score (RSRZ) ) are highlighted in red. **(c, d)** 2|*F*o-*F*c| map contoured 2.0 σ as a side-by-side stereo image (left) or 1.0 σ (right) with a ribbon model of chain E (c) and chain G (d). The built model is generally well fitted to the electron density although residues with high RSRZ values (in magenta) exhibits weaker electron density. Residues with high RSRZ values are located at N/C-terminal regions or the long β2-β3 loop, relatively flexible regions with higher B factors, which presumably causes high RSRZ values.

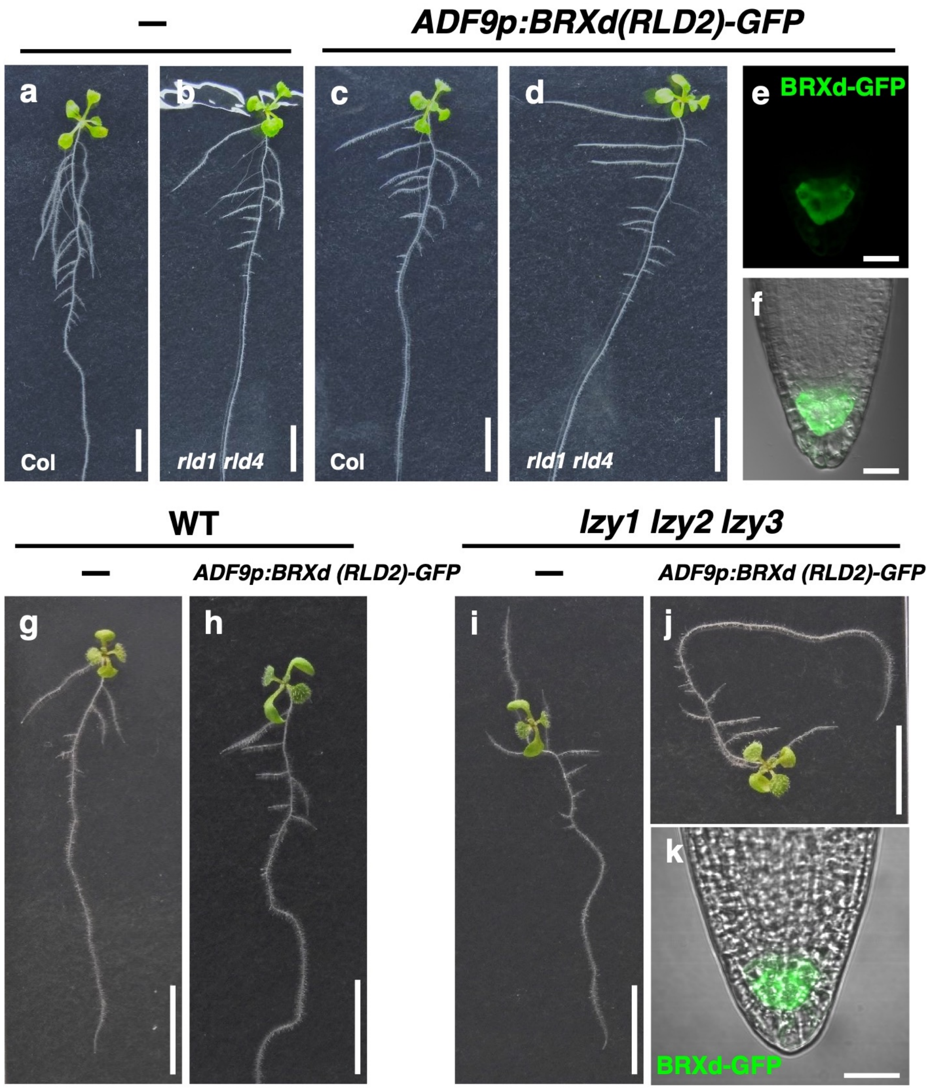


**Supplementary Fig. 16. Root architecture in seedlings statocyte-specifically expressing only RLD2 BRX domain.**

**(a–d)** 11-day-old seedlings of Col (a), *rld1-2 rld4-1* (b), Col harboring *ADF9p:BRXd (RLD2)-GFP* (c), and *rld1-2 rld4-1* harboring *ADF9p:BRXd (RLD2)-GFP* (d). **(e, f)** GFP signal (e) and merged image of GFP and bright field (f) in lateral root of 10-day-old Col harboring *ADF9p:BRXd (RLD2)-GFP*. **(g, h)** 8-day-old seedlings of Col (g) and Col harboring *ADF9p:BRXd (RLD2)-GFP* (h). **(i, j)** 8-day-old seedlings of *lzy1 lzy2 lzy3* (i) and *lzy1 lzy2 lzy3* harboring *ADF9p:BRXd (RLD2)-GFP* (j). **(k)** BRXd (RLD2)-GFP expression in the LR of 8-day-old *lzy1 lzy2 lzy3* seedling harboring *ADF9p:BRXd (RLD2)-GFP.* Scale bars, 1 cm (a–d, g–j), 20 μm (e, f, k).

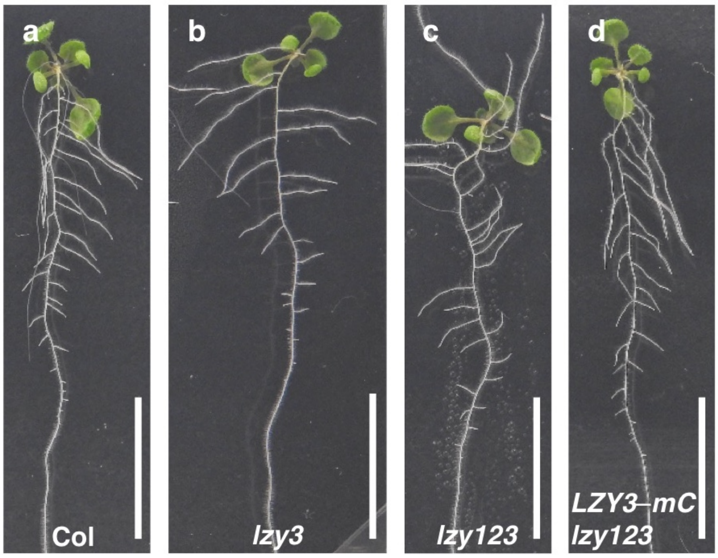


**Supplementary Fig. 17. *LZY3* controls LR GSA.**
**(a–d)** *LZY3* controls GSA in lateral roots. 11-day-old seedlings of Col (a), *lzy3* (b), *lzy1 lzy2 lzy3* (c), and *lzy1 lzy2 lzy3* complemented by *LZY3-mCherry* driven under control of the *LZY3* promoter (d). Scar bars, 2cm.

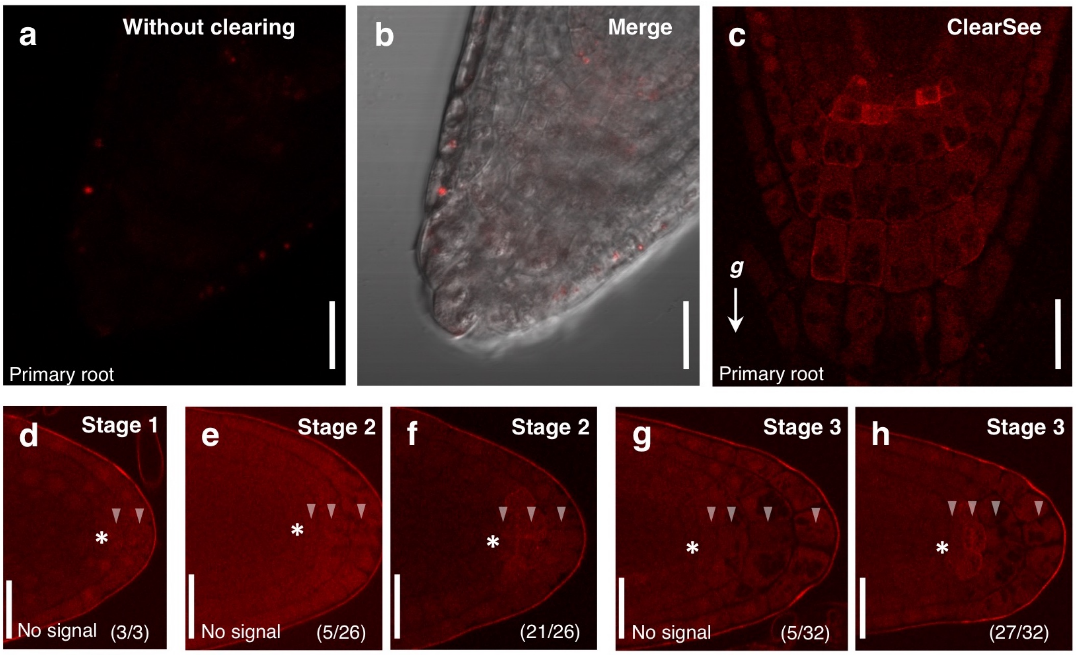


**Supplementary Fig. 18. Localization analysis of LZY3–mCherry using the ClearSee method.**

**(a, b)** No mCherry signals in the primary root of 5-day-old seedling harboring *LZY3p:LZY3-mCherry* without clearing (a). Merged image of fluorescence and bright field (b). **(c)** LZY3-mCherry localization in the primary root of 8-day-old seedling harboring *LZY3p:LZY3-mCherry* with clearing. **(d–h)** LZY3-mCherry localization in the LR of 8-day-old seedlings harboring *LZY3p:LZY3-mCherry* at the stage 1 (d), 2 (e, f), and 3 (g, h) stage of LR development, respectively. Asterisks indicate quiescent center (QC) cells. The LR development was classified into tripartite stages according to the number of columella cell layer between the QC and surface (arrowhead). Stage 1 LRs produce two cell layers between the QC and surface, the stage 2 roots produce three cell layers, and stage 3 produces more than four cell layers. Scale bars, 20 μm.

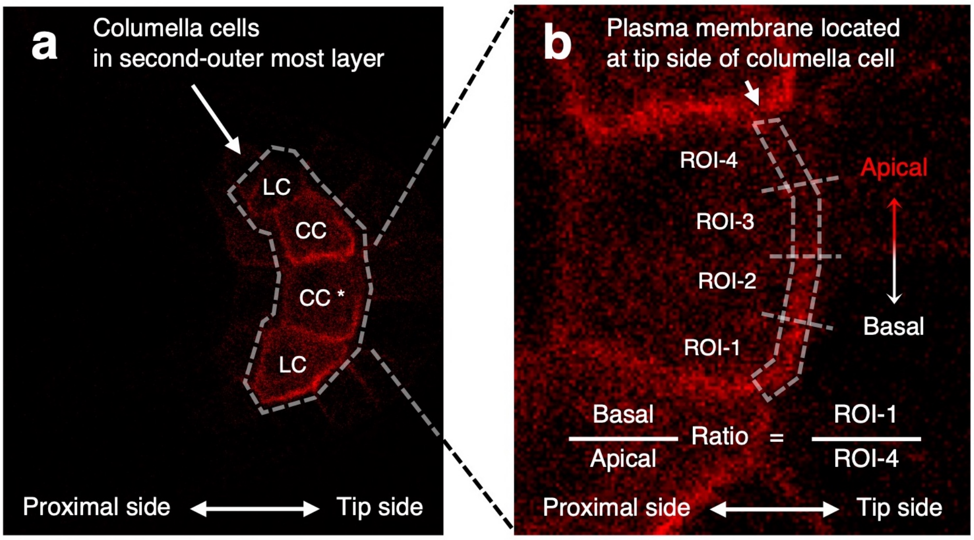


**Supplementary Fig. 19. Measurement of LZY3-mCherry fluorescence intensity**
**(a)** Image showing LZY3-mCherry fluorescence detected in columella cells situated at the second-outer most layer in LR tip. Image shown in Fig. 6a is displayed. Central columella (CC) cells and lateral columella (LC) cells were selected for quantitative analysis of polar localization of LZY3-mCherry. **(b)** Magnified image of the CC cell marked by asterisk in (a). Plasma membrane across the tip side of the cell was selected as region of interest (ROI) for measurement of LZY3-mCherry fluorescence intensity. ROI was equally segmented into 4 compartments from basal side to apical side, giving rise to ROI-1, ROI-2, ROI-3, and ROI-4. ROI-1/ROI-4 ratio of LZY3-mCherry fluorescence intensity was calculated as the Basal/Apical ratio. Double-headed arrows show apical-basal axis of plant body.

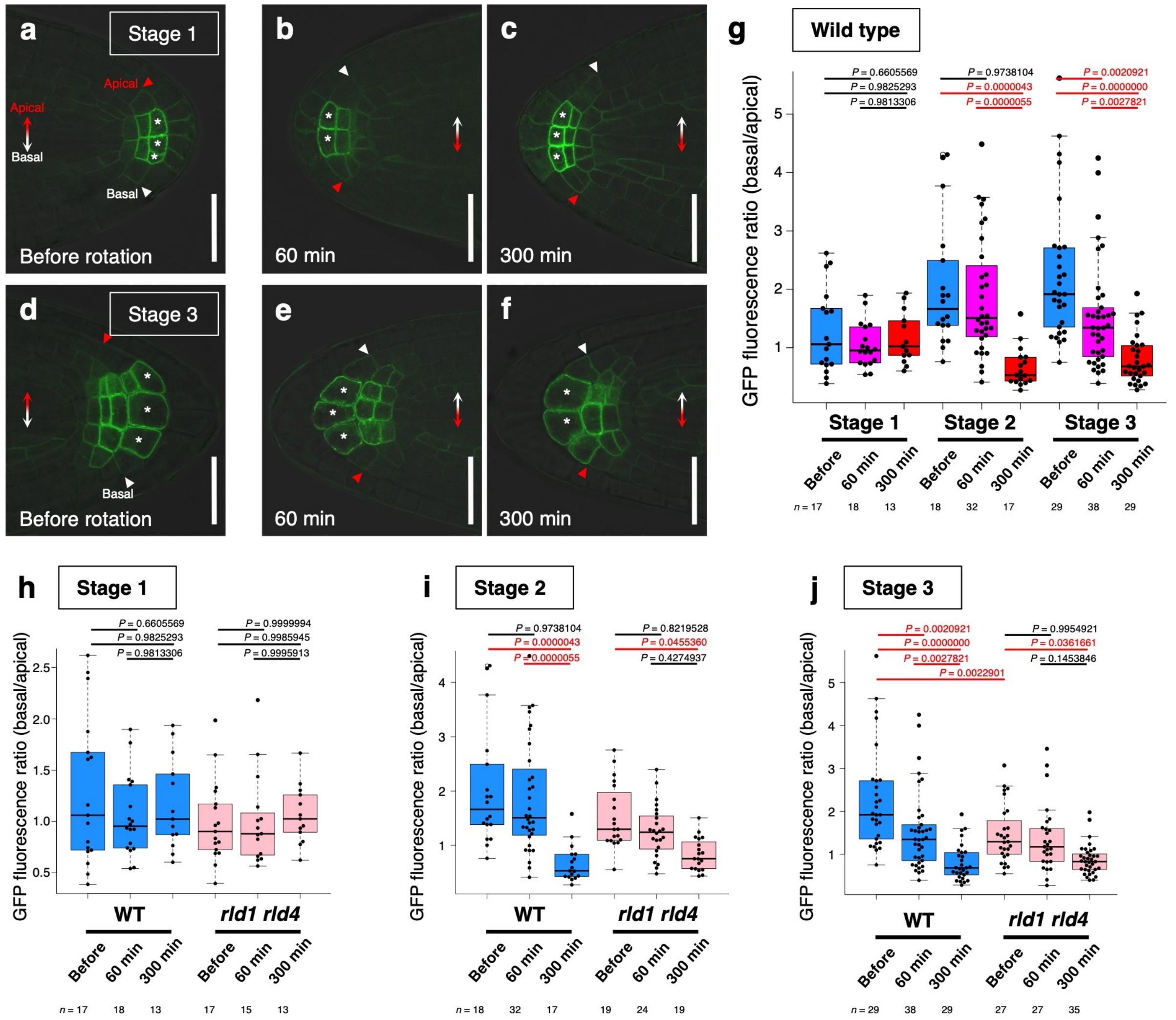


**Supplementary Fig. 20. PIN3 localization in LR tips in response to gravity stimulation.** **(a–f)** The localization of PIN3-GFP in LR tips of 8-day-old seedlings harboring *PIN3p:PIN3-GFP* before rotation (a, d) and at 60 min (b, e), and 300 min (c, f) after 180° rotation at the stage 1 (a–c) and stage 3 (d–f) of LR development. White and red filled arrowheads indicate PIN3-GFP localization in the lateral side of the plasma membrane of columella cells, located basally and apically, respectively, adjacent to central columella cells marked with asterisks at the second-outer most layer in LR tip. Double-headed arrows show apical-basal axis of plant body. Scale bars, 25 µm. **(g)** Asymmetry of PIN3-GFP localization in columella cells was assessed by measurement of GFP fluorescence intensity at lateral plasma membrane domains of columella cells adjacent to central columella cells at the basal flanks compared with those at the apical flanks in lateral root tips of 8-day-old Col seedlings. GFP fluorescence ratio is presented by box lots for PIN3-GFP before rotation (blue) and at 60 min (magenta), and 300 min (pink) after 180° rotation at the stage 1, 2, and 3 of lateral root development. Median and quartile values are provided by the central line and box boundaries. Whiskers show min to max values. *n*, sample number of more than three biologically independent experiments. Statistically significant differences are highlighted in red. Tukey-Kramer method, $P < 0.05$. **(h–j)** Asymmetric localization of PIN3-GFP in LR tips of 8-day-old Col (blue) and *rld1-2 rld4-1* seedlings (pink) harboring *PIN3p:PIN3-GFP* before rotation and at 60 min and 300 min after 180° rotation at the stage 1 (h), stage 2 (i), and stage 3 (j) of LR development. Statistically

significant differences are highlighted in red (Tukey-Kramer method, $P < 0.05$). *n*, sample number of more than three biologically independent experiments. Source data for (g–j) are provided as a Source Data file.

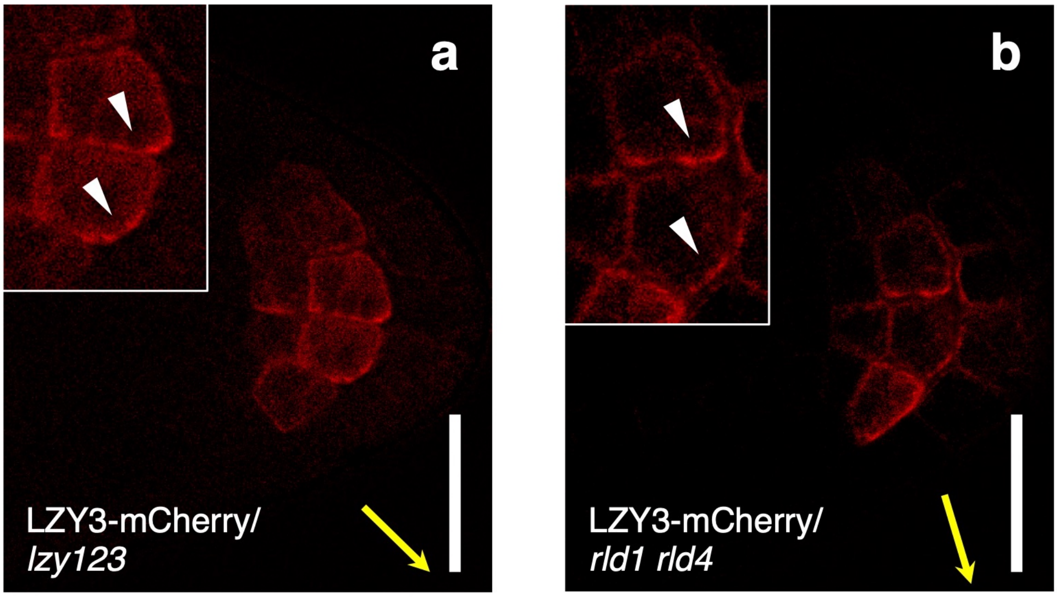


**Supplementary Fig. 21. Localization of LZY3-mCherry in *rld1 rld4* double mutant.**
**(a, b)** The localization of LZY3-mCherry in LR tips of 8-day-old *lzy1 lzy2 lzy3* triple mutant (a) and *rld1-2 rld4-1* double mutant (b) seedlings harboring *LZY3p:LZY3-mCherry*. LZY3-mCherry was detected in the basal side of the plasma membrane in the *rld1-2 rld4-1* double mutant. White arrowheads indicate polarized LZY3-mCherry localization in the plasma membrane. Yellow arrows represent the direction of gravity estimated from the growth orientation of LR tips. Scale bars, 20 μm.

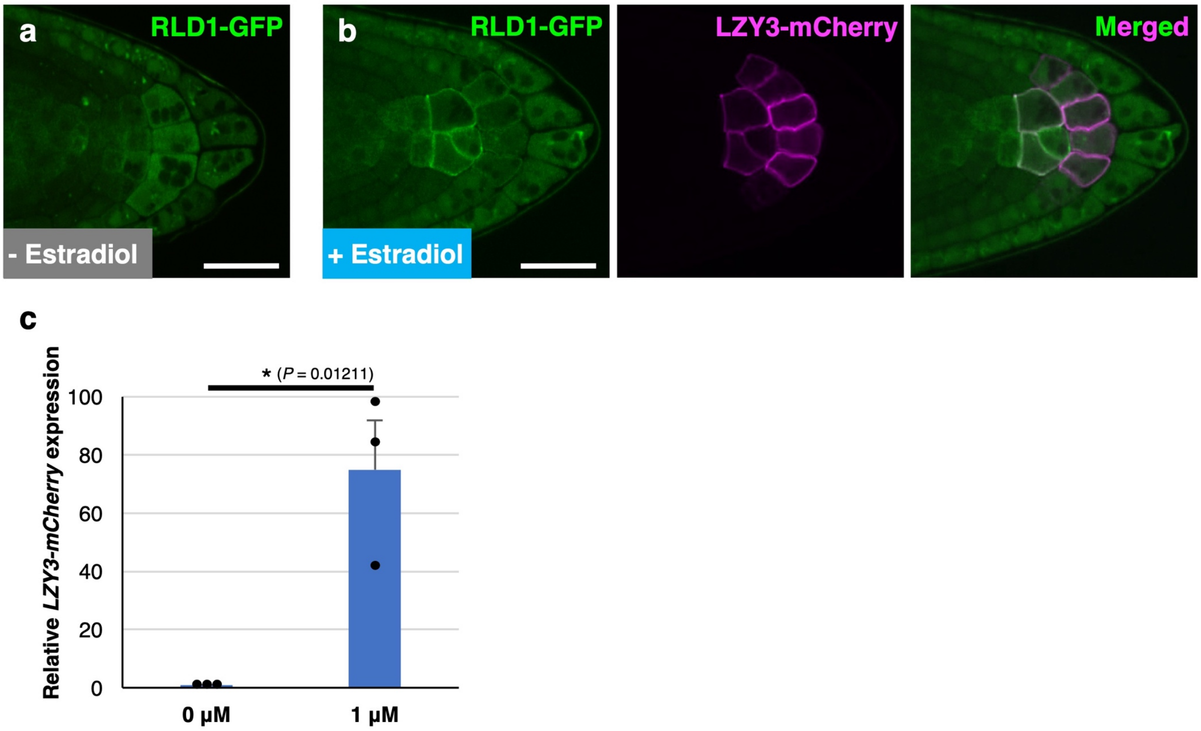


**Supplementary Fig. 22. Overexpressed LZY3-mCherry recruited RLD1-GFP all over the plasma membrane.**
**(a, b)** RLD1-GFP (green) and LZY3-mCherry (red) localization in LRs of 8-day-old seedling harboring *RLD1p:RLD1-GFP* and *ADF9p:XVE>>LZY3-mCherry*, transferred to MS medium plates containing EtOH (a) and 1 µM estradiol (b) at 6 days old. Scale bars, 20 µm. **(c)** Transcript levels of *LZY3-mCherry* in 10-day-old *lzy1 lzy2 lzy3* roots harboring *ADF9p:XVE>>LZY3-mCherry* after treatment with EtOH (0 µM) and Estradiol (1 µM) at 7 days. Expression levels were normalized against expression of the actin gene *ACT8*. Data represent relative values where the relative expression of *LZY3-mCherry* to that of *ACT8* in 0 µM was set as 1. Data consist of three independent samples with SE. Asterisk indicates a significant difference by Student's *t* test (P value < 0.05). Source data for (c) are provided as a Source Data file.

**Supplementary Table 1.**

Candidates of LZY3-interacting protein from Y2H screening

| AGI code | Description in TAIR10 |
|---|---|
| At5g19720 | Regulator of chromosome condensation (RCC1) family with FYVE zinc finger domain-containing protein |
| At5g42140 | Regulator of chromosome condensation (RCC1) family with FYVE zinc finger domain-containing protein |
| At1g76950 | PRAF1, Regulator of chromosome condensation (RCC1) family with FYVE zinc finger domain-containing protein |
| At5g12350 | Regulator of chromosome condensation (RCC1) family with FYVE zinc finger domain-containing protein |
| At3g21190 | MANNAN SYNTHESIS RELATED 1 |

**Supplementary Table 2.**
Candidates of LZY2- and LZY3-interacting protein from LC-MS/MS analyses of immunoprecipitates

| AGI code | Description in TAIR10 | Mascot score | |
|---|---|---|---|
| | | LZY2 | LZY3 |
| AT1G13980 | GNOM | 448 | 27 |
| AT1G23410 | Ribosomal protein S27a / Ubiquitin family protein | 37 | 24 |
| AT1G42970 | glyceraldehyde-3-phosphate dehydrogenase B subunit | 82 | 135 |
| AT1G61520 | photosystem I light harvesting complex gene 3 | 77 | 87 |
| AT1G76950 | PRAF1, Regulator of chromosome condensation (RCC1) family with FYVE zinc finger domain (RLD1) | 504 | 335 |
| AT2G02930 | glutathione S-transferase F3 | 36 | 46 |
| AT2G25730 | zinc finger FYVE domain protein | 21 | 24 |
| AT2G33800 | Ribosomal protein S5 family protein | 49 | 48 |
| AT2G37620 | actin 1 | 100 | 129 |
| AT3G08580 | ADP/ATP carrier 1 | 86 | 74 |
| AT3G12580 | heat shock protein 70 | 231 | 343 |
| AT3G25400 | dCTP pyrophosphatase-like protein | 21 | 20 |
| AT3G46740 | translocon at the outer envelope membrane of chloroplasts 75-III | 65 | 72 |
| AT3G55250 | Encodes a nucleus-encoded protein, Photosystem I Assembly 3 (PSA3), that is required for PSI accumulation. | 17 | 19 |
| AT4G20360 | RAB GTPase homolog E1B | 316 | 211 |
| AT4G32260 | ATPase, F0 complex, subunit B/B', bacterial/chloroplast | 31 | 26 |
| AT5G01530 | light harvesting complex photosystem II | 64 | 68 |
| AT5G02490 | HSP70-2 | 344 | 405 |
| AT5G02500 | HSP70-1 | 624 | 729 |
| AT5G08670 | ATP synthase alpha/beta family protein | 49 | 43 |
| AT5G12350 | Regulator of chromosome condensation (RCC1) family with FYVE zinc finger domain (RLD2) | 349 | 186 |
| AT5G13490 | ADP/ATP carrier 2 | 59 | 69 |
| AT5G19420 | Regulator of chromosome condensation (RCC1) family with FYVE zinc finger domain (RLD3) | 204 | 97 |
| AT5G23060 | calcium sensing receptor | 49 | 58 |
| AT5G35750 | histidine kinase 2 | 34 | 35 |
| AT5G42140 | Regulator of chromosome condensation (RCC1) family with FYVE zinc finger domain (RLD4) | 82 | 41 |

**Supplementary Table 3. X-ray data collection and refinement statistics**

| | RLD2-LZY3 Native | RLD2-LZY3 Se-Met |
|---|---|---|
| **Data collection** | | |
| Space group | *C*2 | $P2_12_12_1$ |
| Cell dimensions | | |
| *a, b, c* (Å) | 89.7, 31.2, 128.9 | 37.8, 55.7, 93.0 |
| α, β, γ (°) | 90.0, 90.0, 91.5 | 90.0, 90.0, 90.0 |
| Resolution (Å) * | 50.0-1.35 (1.37-1.35) | 50.0-1.59 (1.62-1.59) |
| $R_{sym}$* | 6.5 (19.1) | 8.3 (45.6) |
| $I / \sigma(I)$* | 26.1 (3.2) | 17.4 (2.2) |
| Completeness (%)* | 95.4 (90.6) | 98.1 (88.0) |
| Redundancy* | 2.7 (2.1) | 6.6 (4.8) |
| Mosaicity | 0.4-0.89 | 0.16-0.24 |
| | | |
| **Refinement** | | |
| Resolution (Å) | 50.0-1.35 | 50.0-1.59 |
| No. reflections | 75,856 | 26550 |
| $R_{work}$ / $_fR_{ree}$ (%) | 17.7/ 20.1 | 19.1/ 22.4 |
| No. atoms | | |
| Protein | 2684 | 1342 |
| Water | 284 | 160 |
| Ligand | 33 | 30 |
| *B*-factor (Å$^2$) | | |
| Protein | 26.8 | 15.5 |
| Water | 37.0 | 24.7 |
| Ligand | 45.7 | 31.4 |
| R.m.s. deviations | | |
| Bond lengths (Å) | 0.008 | 0.007 |
| Bond angles (°) | 0.915 | 0.972 |

One crystal was used for each data set. $R_{free}$ was calculate on a random 5% reflections of the data.

* Highest resolution shell is shown in parenthesis.

**Supplementary Table 4. Sequences of primers**

| Primer Name | 5' to 3' |
|---|---|
| LZY3-mCh-qRT-F1 | CAGCTCTCGTTGAAGAAC |
| LZY3-mCh-qRT-R1 | GATGGCCATGTTATCCTC |
| ACT8_qPCR-F | TCAGCACTTTCCAGCAGATG |
| ACT8_qPCR-R | CTGTGGACAATGCCTGGAC |
| rld1-1_1-F | ATATGGTCGCAAAGGGAAGC |
| rld1-1_1-R | CCAGCCATCGATTTTTGAGC |
| rld1-1_2-F | AGCGTTATCTTCGCCCAGAG |
| rld1-1_2-R | GACTGATATCAGAGCCATGC |
| rld1-1_3-F | GCAGCTGAAAGATGCTGTG |
| rld1-1_3-R | TGCGCTATGAGCGACTTGATTG |
| rld1-2_1-F | ATATGGTCGCAAAGGGAAGC |
| rld1-2_1-R | CCAGCCATCGATTTTTGAGC |
| rld1-2_2-F | AGCGTTATCTTCGCCCAGAG |
| rld1-2_2-R | GACTGATATCAGAGCCATGC |
| rld1-2_3-F | GCAGCTGAAAGATGCTGTG |
| rld1-2_3-R | TGCGCTATGAGCGACTTGATTG |
| rld2-1_1-F | GGATCTTAGTAGAGCTGGTC |
| rld2-1_1-R | GCGTTGTCCAGATATGATCC |
| rld2-1_2-F | ACATCTGAAGCTGAGCCATG |
| rld2-1_2-R | TATGTCCATGCATGCTATCC |
| rld2-1_3-F | CCGAGAAGGAATATCAGTCG |
| rld2-1_3-R | AACCATGGCTCGAGGAACTAAC |
| rld2-2_1-F | CCGAGAAGGAATATCAGTCG |
| rld2-2_1-R | AACCATGGCTCGAGGAACTAAC |
| rld2-2_2-F | AGTAGCAATGACAGTTTGCAG |
| rld2-2_2-R | TTGCTGCCTTGCATCTTGTCG |
| rld2-2_3-F | CAATAGTAGTCGTGTCTCACC |
| rld2-2_3-R | TCCCAAATGCAGGTGTCATG |
| rld3-1_1-F | AGTCAGAATCTGCCTTTTGGTC |
| rld3-1_1-R | CCCTTCTGCCATACTTAAGC |
| rld3-1_2-F | CGTGATTGCACGAGGTCTATTG |
| rld3-1_2-R | CCAGAGATGATCCTGGAAAC |
| rld3-1_3-F | GCTTAAGTATGGCAGAAGGG |
| rld3-1_3-R | CGGGTATATGTTCTCGGAC |
| rld3-2_1-F | AGTCAGAATCTGCCTTTTGGTC |
| rld3-2_1-R | CCCTTCTGCCATACTTAAGC |
| rld3-2_2-F | CGTGATTGCACGAGGTCTATTG |
| rld3-2_2-R | CCAGAGATGATCCTGGAAAC |
| rld3-2_3-F | GCTTAAGTATGGCAGAAGGG |
| rld3-2_3-R | CGGGTATATGTTCTCGGAC |
| rld4-1_1-F | AGGAAGCGGACTCTTGACTTG |
| rld4-1_1-R | TCTGATCCAGTTCCTCGTAC |
| rld4-1_2-F | GCTTTGATATCTGGTCAGGCTG |
| rld4-1_2-R | GGTATCCAGTGACTTACATCGGTAC |
| rld4-1_3-F | GACTAATGTGGCTGACTTAC |
| rld4-1_3-R | CACAGATTTCTGGACTTCG |

| | |
|---|---|
| rld4-2_1-F | GGAATCTGCCAAATCAGAAGC |
| rld4-2_1-R | CTCTGCCTCCACTTGAGAAG |
| rld4-2_2-F | GTCGCCAAGAAACACAGATG |
| rld4-2_2-R | GTTGCCACACTTGATCTATCG |
| rld4-2_3-F | CAGGTCAAGGATATAGCTGC |
| rld4-2_3-R | ATGCCGGAGAATGAAGCTCTAC |
