# Supplementary material for: Polar recruitment of RLD by LAZY1-like protein during gravity signaling in root branch angle control
Source: Nat Commun. 2020 Jan 3;11:76. doi: 10.1038/s41467-019-13729-7 (PMC6941992; doi:10.1038/s41467-019-13729-7)
Supplement: Supplementary file 2 — Reporting Summary [file 41467_2019_13729_MOESM2_ESM.pdf]

nature research

Corresponding author(s): Miyo Terao Morita
Last updated by author(s): Nov 11, 2019

Reporting Summary

Nature Research wishes to improve the reproducibility of the work that we publish. This form provides structure for consistency and transparency in reporting. For further information on Nature Research policies, see Authors & Referees and the Editorial Policy Checklist .

Statistics

For all statistical analyses, confirm that the following items are present in the figure legend, table legend, main text, or Methods section.

n/a	Confirmed	
☐	☒	The exact sample size (n) for each experimental group/condition, given as a discrete number and unit of measurement
☐	☒	A statement on whether measurements were taken from distinct samples or whether the same sample was measured repeatedly
☐	☒	The statistical test(s) used AND whether they are one- or two-sided<br>*Only common tests should be described solely by name; describe more complex techniques in the Methods section.*
☒	☐	A description of all covariates tested
☒	☐	A description of any assumptions or corrections, such as tests of normality and adjustment for multiple comparisons
☐	☒	A full description of the statistical parameters including central tendency (e.g. means) or other basic estimates (e.g. regression coefficient) AND variation (e.g. standard deviation) or associated estimates of uncertainty (e.g. confidence intervals)
☒	☐	For null hypothesis testing, the test statistic (e.g. F, t, r) with confidence intervals, effect sizes, degrees of freedom and P value noted<br>*Give P values as exact values whenever suitable.*
☒	☐	For Bayesian analysis, information on the choice of priors and Markov chain Monte Carlo settings
☒	☐	For hierarchical and complex designs, identification of the appropriate level for tests and full reporting of outcomes
☒	☐	Estimates of effect sizes (e.g. Cohen's d, Pearson's r), indicating how they were calculated

Our web collection on statistics for biologists contains articles on many of the points above.

Software and code

Policy information about availability of computer code

Data collection	LC-MS/MS was performed using a HTC-PAL/Paradigm MS4 system coupled to a LTQ-Orbitrap XL (Thermo Fisher Scientific). Confocal images were acquired with commercial, built-in softwares of Olympus (FV1000, fluoview), Zeiss (LSM780, ZEN2012), or Leica (TCS SP8 DLS). X-ray diffraction data were collected on BL41XU, BL44XU beamlines at SPring-8 or BL-1A beamline at the Photon Factory. All data were processed and scaled using HKL-2000.
Data analysis	Structure and sequence data were analyzed with SOLVE/RESOLVE, Coot, PHENIX, Phaser, PDBeFOLD server, CLUSTALW, APBS, PyMOL Molecular Graphics System(Version 1.7), or ORIGINTM. MASCOT server (Version 2.4) was used for peptide identification. Quantification of fluorescence intensities was perfotmed with Image J.

For manuscripts utilizing custom algorithms or software that are central to the research but not yet described in published literature, software must be made available to editors/reviewers. We strongly encourage code deposition in a community repository (e.g. GitHub). See the Nature Research guidelines for submitting code & software for further information.

Data

Policy information about availability of data

All manuscripts must include a data availability statement. This statement should provide the following information, where applicable:
- Accession codes, unique identifiers, or web links for publicly available datasets
- A list of figures that have associated raw data
- A description of any restrictions on data availability

The coordinates and structure factors for the Se-Met-labelled RLD2 V1057M-LZY3 complex and the RLD2-LZY3 complex have been deposited in the Worldwide Protein Data Bank (http://www.rcsb.org) with the accession codes 6L0W and 6L0V, respectively. The dataset identifier is PXD016219 for the mass spectrometry proteomics data. The authors declare that the data supporting the findings of this study are available within the manuscript and its supplementary files or are available from the corresponding author upon reasonable request. Raw data for underlying Figs. 1d-e, 2e, 6e-g, and 7i, Supplementary Figs. 7g-j, 12, 14c, 20g-j, and 22c are provided in the Source Data file.

Field-specific reporting

Please select the one below that is the best fit for your research. If you are not sure, read the appropriate sections before making your selection.

- [x] Life sciences
- [ ] Behavioural & social sciences
- [ ] Ecological, evolutionary & environmental sciences

For a reference copy of the document with all sections, see nature.com/documents/nr-reporting-summary-flat.pdf

Life sciences study design

All studies must disclose on these points even when the disclosure is negative.

Sample size	No statistical methods were used to pre-determine sample size.
Data exclusions	No data were excluded.
Replication	All attempts at replication were successful, except for LC/MS analysis of immunoprecipitates. However, we think the result with a paralogous protein (LZY2) can be a replicate.
Randomization	For confocal imaging, cells or roots were selected randomly and representative cells or roots are shown in the manuscript. Plants used for experiments were randomly selected from each genotype.
Blinding	Blinding is not relevant to this study and no animal or human research participants are involved in this study. Arabidopsis thaliana Columbia accession is a pure strain uses as a model plant.

Reporting for specific materials, systems and methods

We require information from authors about some types of materials, experimental systems and methods used in many studies. Here, indicate whether each material, system or method listed is relevant to your study. If you are not sure if a list item applies to your research, read the appropriate section before selecting a response.

Materials & experimental systems

n/a	Involved in the study
[ ]	[x] Antibodies
[ ]	[x] Eukaryotic cell lines
[x]	[ ] Palaeontology
[x]	[ ] Animals and other organisms
[x]	[ ] Human research participants
[x]	[ ] Clinical data

Methods

n/a	Involved in the study
[x]	[ ] ChIP-seq
[x]	[ ] Flow cytometry
[x]	[ ] MRI-based neuroimaging

Antibodies

Antibodies used	µMACS Anti-GFP MicroBeads
Validation	µMACS Epitope-Tagged Protein Isolation Kit (Miltenyi Biotec)

Eukaryotic cell lines

Policy information about cell lines

Cell line source(s)	Arabidopsis suspension culture cell. Takeuchi et al., Plant J. 2000, 23:517-525
Authentication	not applicable
Mycoplasma contamination	not applicable
Commonly misidentified lines (See ICLAC register)	not applicable
